# Genomic features of *“Candidatus* Venteria ishoeyi”, a new sulfur-oxidizing macrobacterium from the Humboldt Sulfuretum off Chile

**DOI:** 10.1371/journal.pone.0188371

**Published:** 2017-12-13

**Authors:** Alexis Fonseca, Thomas Ishoey, Carola Espinoza, Danilo Pérez-Pantoja, Antonio Manghisi, Marina Morabito, Alexis Salas-Burgos, Víctor A. Gallardo

**Affiliations:** 1 Department of Pharmacology, University of Concepcion, Concepcion, Chile; 2 Department of Oceanography, University of Concepcion, Concepcion, Chile; 3 Independent consultant, Encinitas, California, United States of America; 4 College of Ocean Science and Resources, Institute Marine Affairs and Resource Management, National Taiwan Ocean University, Keelung, Taiwan; 5 Programa Institucional de Fomento a la Investigación, Desarrollo e Innovación, Universidad Tecnológica Metropolitana, San Joaquin, Santiago, Chile; 6 Department of Chemical, Biological, Pharmaceutical and Environmental Sciences, University of Messina, Messina, Italy; The University of Akron, UNITED STATES

## Abstract

The Humboldt Sulfuretum (HS), in the productive Humboldt Eastern Boundary Current Upwelling Ecosystem, extends under the hypoxic waters of the Peru-Chile Undercurrent (*ca*. 6°S and *ca*. 36°S). Studies show that primeval sulfuretums held diverse prokaryotic life, and, while rare today, still sustain species-rich giant sulfur-oxidizing bacterial communities. We here present the genomic features of a new bacteria of the HS, “*Candidatus* Venteria ishoeyi” (“*Ca*. V. ishoeyi”) in the family *Thiotrichaceae*.Three identical filaments were micro-manipulated from reduced sediments collected off central Chile; their DNA was extracted, amplified, and sequenced by a Roche 454 GS FLX platform. Using three sequenced libraries and through *de novo* genome assembly, a draft genome of 5.7 Mbp, 495 scaffolds, and a N50 of 70 kbp, was obtained. The 16S rRNA gene phylogenetic analysis showed that “*Ca*. V. ishoeyi” is related to non-vacuolate forms presently known as *Beggiatoa* or *Beggiatoa*-like forms. The complete set of genes involved in respiratory nitrate-reduction to dinitrogen was identified in “*Ca*. V. ishoeyi”; including genes likely leading to ammonification. As expected, the sulfur-oxidation pathway reported for other sulfur-oxidizing bacteria were deduced and also, key inorganic and organic carbon acquisition related genes were identified. Unexpectedly, the genome of “*Ca*. V. ishoeyi” contained numerous CRISPR repeats and an I-F CRISPR-Cas type system gene coding array. Findings further show that, as a member of an eons-old marine ecosystem, “*Ca*. V. ishoeyi” contains the needed metabolic plasticity for life in an increasingly oxygenated and variable ocean.

## Introduction

Below the oxygen-deficient waters of the Peru-Chile Undercurrent, between central Peru (*ca*. 6°S) and central Chile (*ca*. 36°S), the Humboldt Sulfuretum (HS), a sulfur compound- and macro-, megabacteria-rich biotope has been described [1–5]. Of varying morphology, the diameter of the filamentous macrobacteria typically range from ca. 1 μm to *ca*. 10 μm, and their lengths from around 10 μm to mostly several hundreds or even several thousands of micrometers. The megabacteria in turn, are defined as having a diameter greater than around 10 μm, most generally exhibiting a vacuolar system, and lengths that can reach several hundred to several thousands, and even millimeters. This classification of large filamentous bacteria, while ecologically useful, probably reflects their adaptation and evolution to an ocean evolving from fully or mostly anoxic to fully or mostly oxic [2]. Presenting results from a study on a member of the macrobacteria group is the objective of the present work.

While the term sulfuretum stems from stagnant fresh-water systems [6], recent study suggest that this type of benthic habitat and its typical biological complements, *i*.*e*., a highly diverse community of big filamentous and spherical non-photosynthetic sulfur-oxidizing bacteria [5], and small Archaea, might have been the only or the dominant types of life in primeval ocean bottoms when free oxygen was lacking or scarce [5, 7]. Paleontological, geological, and geochemical evidence from this habitat and its biota have been reported from the Archaean and Proterozoic Eons [8–10], and from late Miocene (Tertiary), Italian mudstone beds [11]. We further posit that such evidence might still be present in the late Jurassic (Oxfordian), “proto-Humboldt Sulfuretum”-derived fossil-bearing deposits in northern Chile’s “Cordillera Domeyko” [12]. Further, the recently described early occupation of new substrate created by a submarine volcanic eruption, of the closely related *Thiotrichaceae* “*Ca*. Thiolava veneris”, could also be interpreted as evidence of biotic linkages between present sulfur bacteria and primeval benthic ecosystems [13].

Following the probable course of evolution, filamentous sulfur oxidizing macrobacteria oxidize sulfur directly using environmental nitrate [14–16], while sulfur oxidizing megabacteria do it through their highly concentrated vacuole-stored nitrate. Moreover, most big sulfur-oxidizing bacteria utilize stored intracellular elemental sulfur, and in some of them, phosphorus accumulations as polyphosphate have been reported [17–19].

When conditions are suitable, *i*.*e*., the environment offers the right levels of electron donors and electron acceptors [20–23], mega- and macrobacteria form extensive and massive mats [4, 24].

According to the currently accepted classification system all discovered colorless sulfur-oxidizing macro- and megabacteria e.g., [2, 25, 26], including the organisms of the present study, are classified in the family *Thiotrichaceae* [27], where the founder genus was *Beggiatoa* [28].

As predicted [29] on the potential extremes in terms of morphology and physiology of large colorless sulfur-oxidizing bacteria, they recently underwent a major taxonomic overhaul affecting two of its classical filamentous, multicellular genera, *i*.*e*., *Beggiatoa* and *Thioploca*, and proposing several new *candidatus* lineages [25]. At the same time, several drafts and some finished genomes of colorless sulfur-oxidizing, macro- and megabacteria, both from fresh- and seawater, as well as a couple of spherical representatives are available in the literature. Among the marine megabacteria and spherical, these include “*Ca*. Isobeggiatoa divolgata”, “*Ca*. Parabeggiatoa communis” [30], “*Ca*. Maribeggiatoa vulgaris” [31, 32], *Thioploca ingrica* [33], *“Ca*. Thiomargarita nelsonii Thio36*”* [34], *“Ca*. Thiomargarita nelsonii Bud S10” [35], and “*Ca*. Marithrix sp.” [36]. Among the macrobacteria, genomes became available for the narrow non-vacuolated fresh-water strain *Beggiatoa alba* B18LD (BioProject PRJNA224116 at the JGI; collected from fresh-water covered rice field, Louisiana, U.S.A.), and for *Beggiatoa leptomitiformis* D-402T (isolated from a domestic sewage polluted stream) [37], as well as the narrow non-vacuolated marine strain *Beggiatoa* sp. 35Flor (MG Genome ID 2606217769; collected from a microbial consortium of a black band disease in an epibenthic scleractinian coral in the Florida Keys). More recently, the partial genome of the narrow sheathed *Thiotrichaceae* macrobacterium “*Candidatus* Thiolava veneris” from a new volcanic sublittoral habitat (130 m deep) became available [13].

We here present the new filamentous sulfur-oxidizing bacteria “*Candidatus* Venteria ishoeyi” (“*Ca*. V. ishoeyi”), member of the highly diversified microbial community from the HS [5].

Seven filaments were initially isolated through micromanipulation from highly reduced soft HS-sediment samples, collected in 35 m depth, near the mouth of the Bay of Concepción, in central Chile. Next, the DNA from three out of seven filaments, evaluated as belonging to the same species (“*Ca*. V. ishoeyi”), were amplified by multiple displacement amplification (MDA) and sequenced through the Roche 454 GS FLX sequencing platform, to finally assemble these libraries in a single draft genome.

To uncover the main genomic features of the “*Ca*. V. ishoeyi” filaments, the potential for nitrate-reducing, sulfur-oxidizing metabolism, phosphorus, and carbon acquisition, as well as, the presence of CRISPR-Cas system, and gene clusters potentially involved in the biosynthesis of secondary metabolites, were assessed.

## Materials and methods

### Sampling

**Fig 1:**
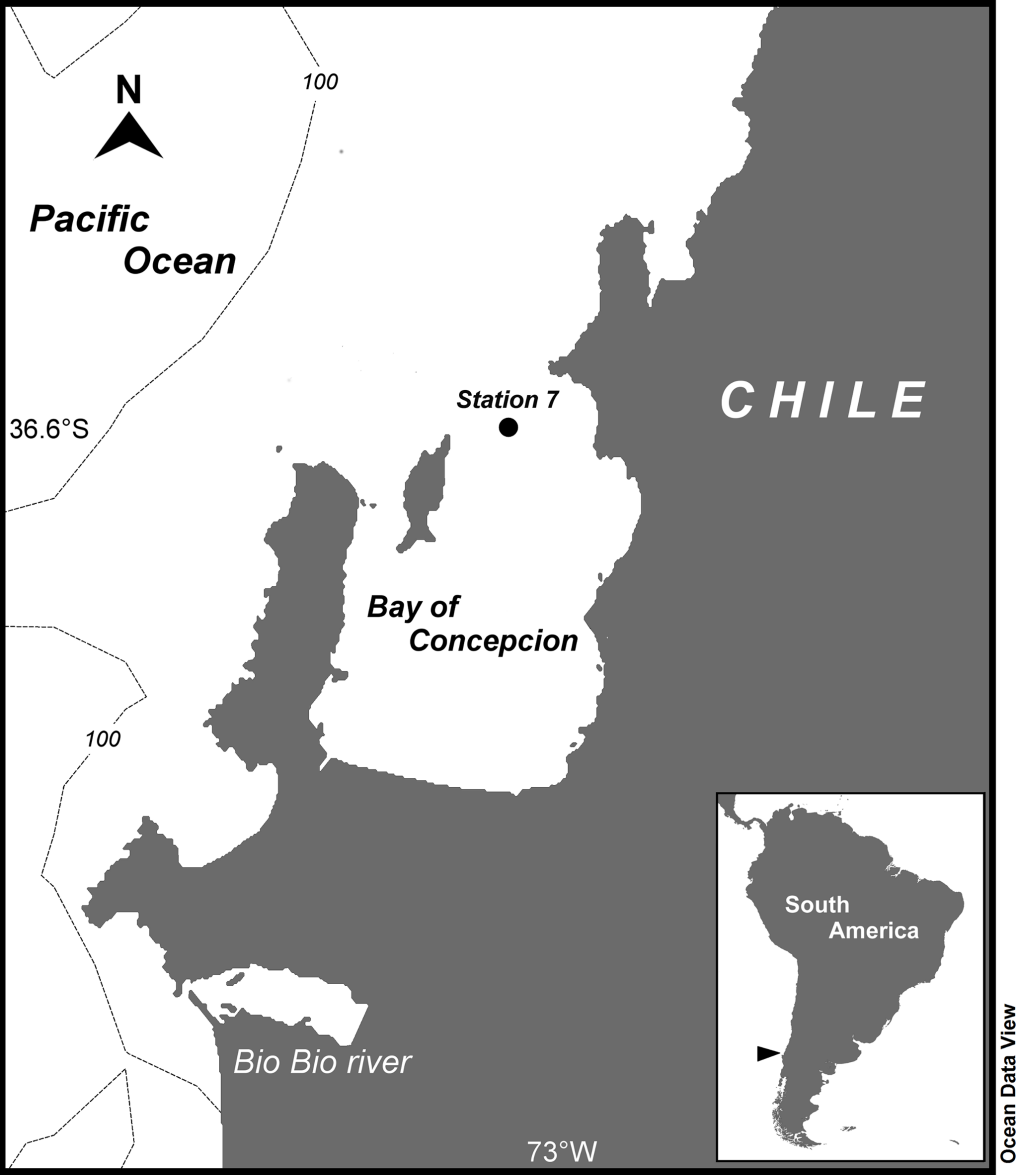
Sampling site. Map shows the location of sampling Station 7 (35 m depth) at the margins of the HS in the mouth of the Bay of Concepcion, central Chile.

Sampling was carried out on December 13, 2008, a normal El Niño Southern Oscillation (ENSO) cycle year, *i*.*e*., a non-warm El Niño and non-extremely cold La Niña year, at the benthic Station 7 (Lat. -36.64, Long. -73.04) [3] located near the mouth of the Bay of Concepcion (central Chile) at 35 m depth (Fig 1). Sediments were sampled from the 8.2 m R/L “Otilia” motor-whale boat with an in-house built mono-coring device equipped with a 1 m long, 5 cm in diameter sampling Plexiglas tube (Note: being a public space, special permissions was not required for sampling in this area. Moreover, the sampling area is not inhabited for protected species and we have not used sampling or experimental methods that endangered the place or the species inhabit there).

During a normal cold ENSO phase at the sampling site, dissolved oxygen near the bottom varies between *ca*. 0.2 mL L^-1^ during mid-summer and 1.3 mL L^-1^ in late winter. Over the shallow shelf at the end of the austral summer (March) maximum and minimum temperatures at surface and at 50 m depth are typically of *ca*. 14°C and 11°C, respectively. At the bottom, redox measurements showed highly reduced conditions with the abundant presence of hydrogen sulfide felt organoleptically [3, 4]. Detailed hydrographic conditions data and information on the coastal sea dynamics of the area resulting from two summer cruises of the “*Thioploca*-Chile 1994 Expedition”, carried out in cooperation with the Max Planck Institute for Marine Microbiology, Bremen, Germany; and the Chilean Navy are available [3, 38].

### Filament micromanipulation and amplification-sequencing of DNA

**Fig 2:**
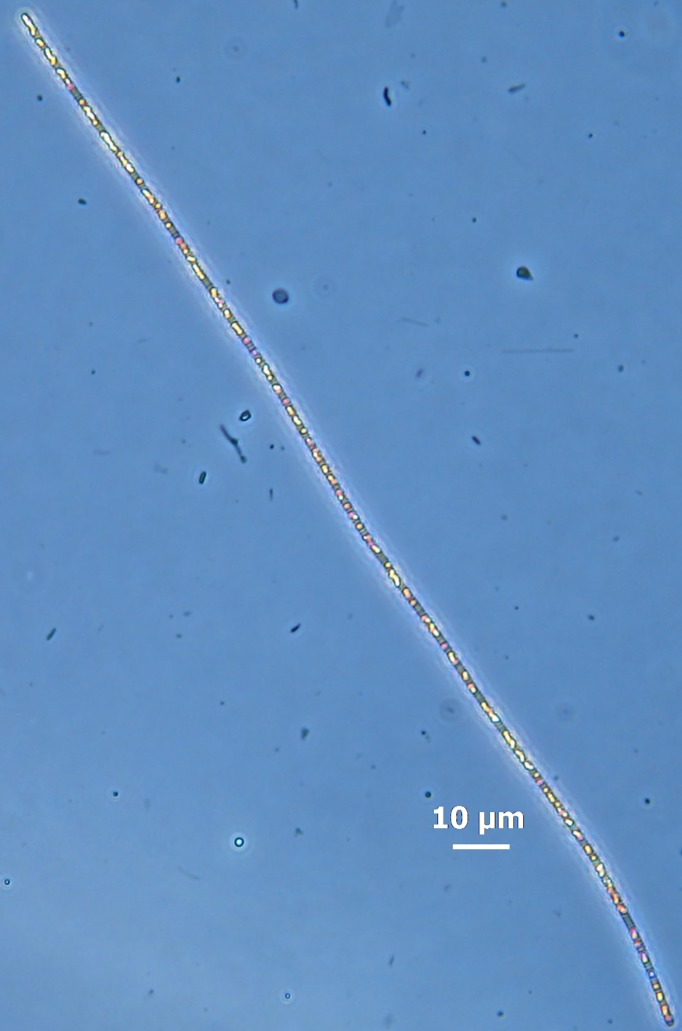
Microphotograph of the MDA1 macrobacteria filament which was later isolated and its whole genome amplified. Isolations were done using microcapillaries approximately 10 μm in diameter **(**Photo CE).

Micromanipulation of seven single narrow, non-vacuolated filaments (Fig 2) from freshly collected samples was performed following Ishoey’s [39] method, and their whole genome was amplified. Genome amplifications were carried out during two separate sessions (Dec. 2008 and Feb. 2009). Firstly, samples were diluted in sterile filtered seawater to reduce cell densities. This step was followed by isolation, micromanipulation, and washing/cleaning of selected filaments in sterile filtered seawater. Next, filaments were transferred to 0.5 or 1 μL sterile PBS in a 200 μL PCR tube for whole genome amplification. Amplifications were done using the GenomiPhi HY kit (GE Healthcare) by the alkaline lysis method and were terminated after 6 hr at 30°C. WGA products were diluted 2-fold in TE-buffer (stored at -20°C) and a 20-fold dilution was prepared as working solution for PCR analysis and quantification. The purity of the amplified DNA was examined by PCR/sequencing of the 16S rRNA gene using universal primers 27F (5’-AGA GTT TGA TCM TGG CTC AG-3’) and 1492R (5’-CGG TTA CCT TGT TAC GAC TT-3’) and sequenced by Sanger sequencing. PCR products were visualized by agarose gel electrophoresis (81%) E-gels (Invitrogen) with a low DNA mass 2kb ladder. MDA and corresponding full length (~1.5 kbp) 16S rDNA PCR products are shown in S1 Fig. Analysis of the 16S rRNA sequences confirmed that MDA1, MDA2 and MDA5 (S1 Table) contained identical species of bacteria. In consequence, MDA1 and MDA2 were selected for genome analysis by sequencing of 3kb mate pair library, and MDA5 for single-end library through the Roche 454 GS FLX sequencing platform. Next, in order to assess the reads identity level, the three libraries were aligned using the software bbmap from BBTools suite tools (http://jgi.doe.gov/data-and-tools/bbtools/), which showed that ~94% of the reads aligned effectively. Thus, based on the previous results, the MDA1, MDA2, and MDA5 library, were pooled to rebuild the draft genome of “*Ca*. V. ishoeyi”.

Reads and annotation of the draft genome assembly are available through the PRJEB15360 Project id of The European Nucleotide Archive (ENA). Filament micromanipulation, amplification, and DNA sequencing were performed at Synthetic Genomics Inc. (SGI), La Jolla, CA, USA.

### Genome assembly and annotation

Read sequences were converted and divided (if mate pair reads were detected) using sffToCA, a subroutine of the Celera (WGS) assembler [40]. Read quality control was performed through the FastQC (www.bioinformatics.babraham.ac.uk/projects/), which provides for a fast and easy display of quality. Then, low-quality reads and noncoding DNA fragments were filtered and removed through Prinseq-lite software (http://prinseq.sourceforge.net).

*De novo* genome assembly was carried out using Celera assembler on a Rocks cluster of 64 cores. The main assembly metrics were estimated by a custom python script and scaffolds >1000 bp were selected for gene prediction.

In order to determine possible contamination, the draft genome obtained was subjected to binning analysis using the MetaWatt software [41]. Moreover, the Amphora2 software [42] was used to evaluate the presence of 32 bacterial marker genes. The genome completeness of “*Ca*. V. ishoeyi” was assessed using the number of identified tRNAs, in reference to the number of tRNAs present in the complete genome of *Thioploca ingrica* (44 tRNAs—1 contig) [33] and *Beggiatoa leptomitiformis* (47 tRNAs—1 contig) [37], next to the presence of 139 single-copy genes [43].

Coding DNA sequences (CDSs) were predicted using a combination of the RAST platform [44] and the stand-alone Prokka program (rapid prokaryotic genome annotation) [45]. Ribosomal RNA genes were identified using Barrnap version 3 (Basic Rapid Ribosomal RNA Predictor) (http://www.vicbioinformatics.com/software.barrnap.shtml), which uses the HMMER tool (http://hmmer.org/). Additional sequence analyses were performed through the Pfam database [46], BLASTN and BLASTP searches using non redundant databases. Also, to explore the presence of gene clusters involved in biosynthesis of secondary metabolites, the antiSMASH stand-alone software version 3.0 [47–49] and the NapDos platform [50] to detect C- and KS- domains, were used. Additionally, the identification of CRISPR repeats was carried out by the CRISPRFinder tool [51]. The assembly visualization was made with the Hawkeye tool from the AMOS open-source project Version 3.1.0 [52] and Artemis [53]. The circular representation of the draft genome was built through the Circos software [54]. Finally, CDSs were considered as protein-coding genes if they agreed with the following criteria in the top hit of the BLASTP analysis: E-value <1e - 8 and sequence identity >30%.

### Phylogenetic analysis

After genome assembly, the 16S rRNA gene (MBHS_03239–1,537 bp in length) was identified using Barrnap. The phylogenetic tree was built using the 16S rRNA gene sequence identified in the genome of “*Ca*. V. ishoeyi”, together with 16S rRNA gene sequences of 37 representative sulfur-oxidizing bacteria, obtained from NCBI. Sequences were aligned using Clustalw2 (http://www.clustal.org/clustal2/) and the phylogenetic tree was built through the bayesian approach with MrBayes tool [55], using Markov chain Monte Carlo (MCMC) method to estimate the *a posteriori* probability, one million of generations, GTR (General Time Reversible) as substitution model (substitution rate = 6), highest likelihood, and discarding 25% of the sampled trees. The 16S rRNA gene sequences of *Thiothrix nivea* (L40993), *Leucothrix mucor* (X87277), and *Achromatium oxaliferum* (L42543), were used as outgroup. The *a posteriori* probability was represented in percentage from 0 to 1 over the nodes.

The amino acid sequences were aligned using mafft v7.221 [56], and the phylogenetic tree was built with the same method used for the 16S rRNA gene sequences, but protein as nucmodel and 500.000 generations.

## Results and discussion

### Metrics and general features of the draft genome of “*Ca*. V. ishoeyi”

The sequencing of two 3 kbp mate pair libraries and a single-end library rendered 377 Mbp in 1,175,553 raw reads with 240 bp in average for mate pair libraries and 450 bp for the single-end library. After the preprocessing stage, 244,880 and 245,912 reads for the 3 kbp mate pair libraries and 476,770 reads for the single-end library were obtained.

*De novo* assembly was applied to obtain a draft genome, through the Celera assembler, taking as input two mate-pair libraries and a single-end library. A genome size of 5.7 Mbp (Table 1), 719 contigs in 495 scaffolds, a N50 of 30.9 kbp for contigs and 70.2 kbp for scaffold, 41.1% of GC content, and 34X of coverage, was produced.

**Table 1:** Main genomic features among some representative sulfur-oxidizing bacteria and the draft genome of “*Ca*. V. ishoeyi”.

Feature	“*Ca*. V. ishoeyi” (ma)	*Beggiatoa* sp. 35Flor (ma)	*Beggiatoa alba* B18LD (ma)	“*Ca*. Isobeggiatoa divolgata” (me)	“*Ca*. Maribeggiatoa sp.” (me)	“*Ca*. Thiomargarita nelsonii Thio36” (sph)
Genome size (Mbp)	5.7	4.0	4.3	7.6	4.8	5.3
Contigs	719	291	21	6,769	822	3,613
N50 (kbp)	30.9	n.d.	347	2.2	15.4	1.8
Max.contig length (kbp)	113	138	500	19	71	14
GC (%)	41.1	38.5	40.0	38.5	38.2	42.0
tRNAs	41	38	46	45	46	23
CDSs coding protein with known function	2,828	2,746	2,867	3,414	2,926	3,486
Genome completeness (%)*	95.0	97.8	100	98.5	98.5	70.0

* Based on 139 single-copy genes published by Campbell *et al*. (2013) [43].

ma = macrobacteria; me = megabacteria; sph = spherical bacterial; n.d. = no data. Table modified from Winkel *et al*. (2016) [34].

The genome size (5.7 Mbp) and GC content (41.1%) of “*Ca*. V. ishoeyi” are in the range of other drafts and complete genomes from related bacteria (Table 1). For example the filamentous megabacteria “*Ca*. Isobeggiatoa divolgata“, a genome size of 7.6 Mbp and a GC content of 38.5% have been reported [30]. For the finished genome of the freshwater filamentous macrobacteria *Thioploca ingrica* a genome size of 4.8 Mbp and a GC content of 41.21% is reported [33], close to the draft genome of the filamentous megabacteria “*Ca*. Maribeggiatoa sp.” (4.8 Mbp—GC = 38.5%) [31, 32], and to the finished genome of the filamentous macrobacteria *Beggiatoa leptomitiformis* (4.3 Mbp–GC = 40.4%) [37]. The GC content for the bacteria in Table 1, ranges from 38.5% in the filamentous megabacteria “*Ca*. Maribeggiatoa sp.” [31, 32] to 42% in the spherical “*Ca*. Thiomargarita nelsonii Thio36” [34], whereas the macrobacteria “*Ca*. V. ishoeyi” has a GC content of 41.1%.

**Fig 3:**
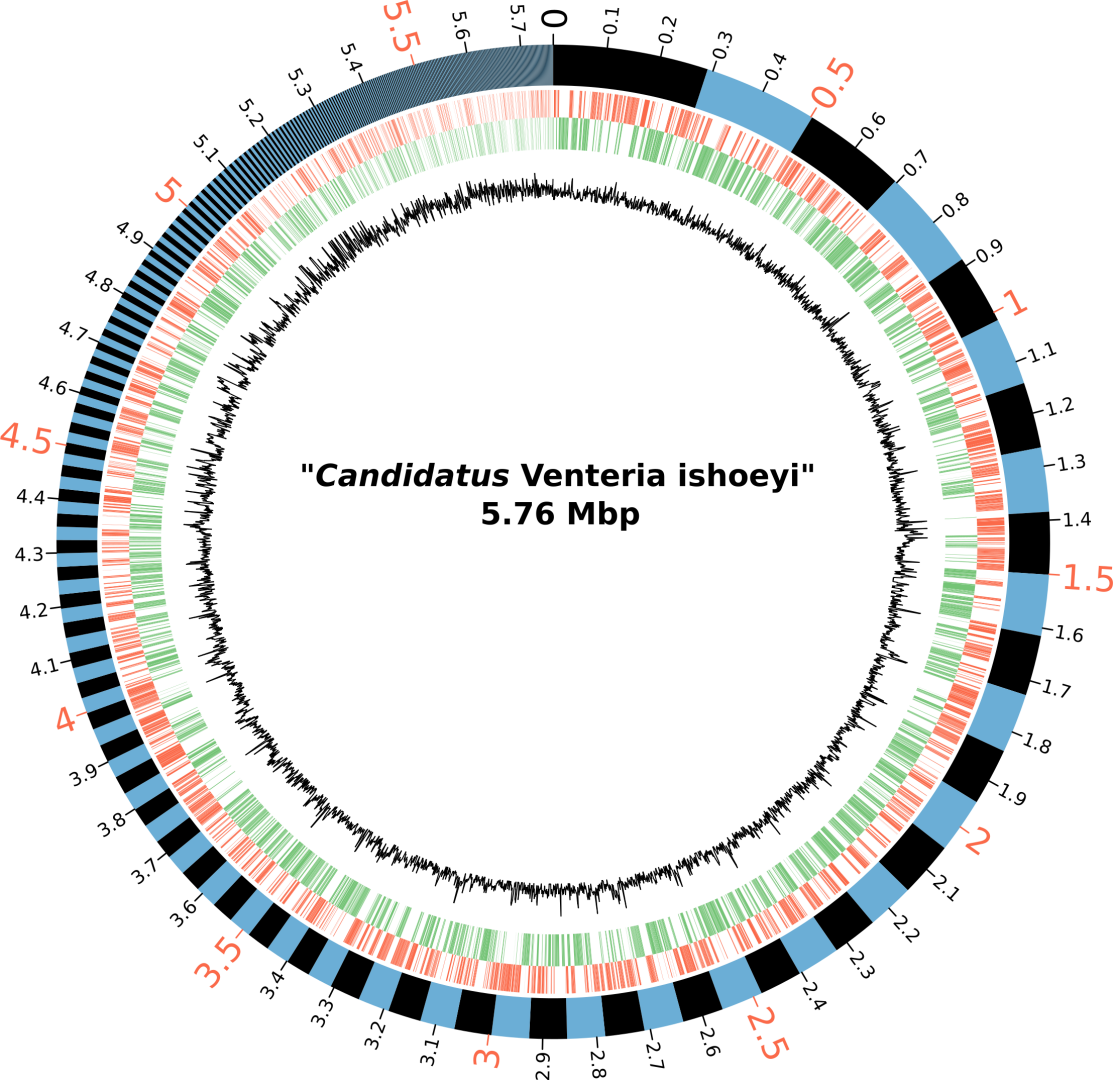
Circular representation of the draft genome of “*Ca*. V. ishoeyi”. Moving out from the innermost circle: the black circle describes the GC% content in a window = 1000 bp; the green circle represents the CDSs of the antisense DNA strand, and the red circle the CDSs of the sense DNA strand; the outermost circle shows all scaffolds, each one alternating between blue and black.

In the draft genome of “*Ca*. V. ishoeyi” (Fig 3), 4,767 coding sequences (CDSs), 5 rRNA (one 16S, one 23S, and three 5S), 41 tRNA, and one tmRNA, were identified. Out of 4,767 CDSs, 1,939 corresponded to hypothetical proteins leaving 2,828 CDSs encoding for proteins with a known function. Three sequences of 5S rRNA, each one of 109 bp in length, were identified, a feature that may respond to the nature of the assembly, which resulted from pooling the DNA of three different “*Ca*. V. ishoeyi” filaments. The 23S rRNA gene was broken in two separated segments of *ca*. 1,600 bp in a contiguous region (MBHS_03236–37).

The genome completeness of “*Ca*. V. ishoeyi” was evaluated by the presence of the single-copy genes [43], including all ribosomal proteins; and the number of tRNAs, using as reference the complete genomes of *Thioploca ingrica* (44 tRNAs—1 contig) [33], and of *Beggiatoa leptomitiformis* D-402 (47 tRNAs—1 contig) [37]. Results show the presence of 41 tRNAs in “*Ca*. V. ishoeyi”, with a level of completeness between 87% (compared with *Thioploca ingrica*), and 94% (compared with *Beggiatoa leptomitiformis* D-402). Furthermore, 132 single-copy genes were identified, thus achieving a 95% of completeness for draft genome of “*Ca*. V. ishoeyi”. Although the completeness is higher than reported for “*Ca*. Thiomargarita nelsonii Thio36” (70%) [34], and “*Ca*. Thiomargarita nelsonii Bud S10” (89.8%) [35], it is lower than those reported for “*Ca*. Isobeggiatoa divolgata” (98.5%) [30], “Ca. Maribbegiatoa sp.” (98.5%) [31], *Beggiatoa* sp. 35Flor (97.8%) (IMG Genome ID 2606217769), *Beggiatoa alba* B18LD (100%) (BioProject PRJNA224116), and *Thioploca ingrica* (100%) [33] (Table 1). Besides, the number of tRNAs quoted above ranges from 23 tRNAs in “*Ca*. Thiomargarita nelsonii Thio36” to 47 in *Beggiatoa leptomitiformis* D-402. Thus, the genome completeness of “*Ca*. V. ishoeyi” is high, being close to the finished genomes of other related *Thiotrichaceae*.

### 16S rRNA phylogeny

**Fig 4:**
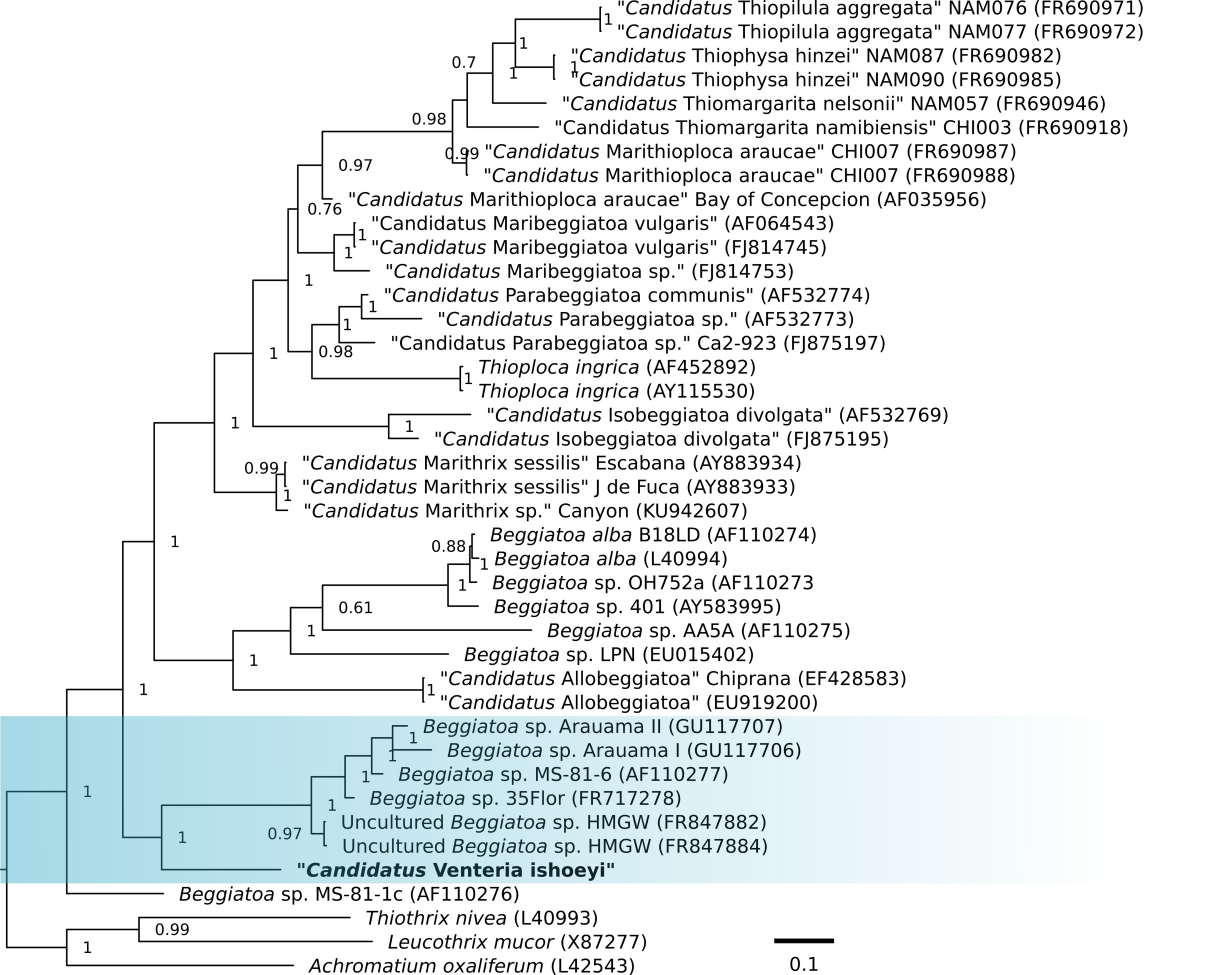
Phylogenetic tree based on 16S rRNA gene sequences. Blue rectangle depicts the clade for “*Ca*. V. ishoeyi”. In bold is shown the position for “*Ca*. V. ishoeyi” into the clade, and the numbers next to the nodes indicates the *a posteriori* probability in percentage of 0 to 1. The tree was calculated using Bayesian approach with MrBayes tool.

The 16S rRNA gene is the most utilized molecular marker for bacteria identification since it possesses a constant functionality and it is thus considered a valid molecular chronometer, essential to inferring precise phylogenetic relationships among organisms [57–60]. The phylogenetic analysis was made using the 16S rRNA gene sequence identified in “*Ca*. V. ishoeyi” and the 16S rRNA gene sequences of 37 representative sulphur-oxidizing colorless bacteria. Further, three sequences belonging to *Thiothrix nivea* (L40993), *Leucothrix mucor* (X87277), and *Achromatium oxaliferum* (L42543), were used as outgroup. The phylogenetic tree (Fig 4) shows that “*Ca*. V. ishoeyi” affiliates to the root of the tree in a monophyletic clade composed of the marine *Beggiatoa* sp. MS-81-6 [61], *Beggiatoa* sp. Arauama I, *Beggiatoa* sp. Arauama II, from hypersaline coastal lagoon Araruama [62], the marine *Beggiatoa* sp. 35Flor [63], the uncultured *Beggiatoa* sp. HMW from the Hakon Mosby mud volcano area [64], and close to *Beggiatoa* sp. MS-81-1c [61]. Within the clade, “*Ca*. V. ishoeyi” shares between 90% and 91% sequence identity with the other 16S rRNA gene sequences, and have even less sequence identity with narrow freshwater *Beggiatoa* or the wide, vacuolated marine sulfur-oxidizing bacteria.

The species close to “*Ca*. V. ishoeyi” are “narrow” marine or from hypersaline systems, non-vacuolate macrobacteria from different sources, *i*.*e*., *Beggiatoa* sp. Araruama^1^ II and *Beggiatoa* sp. Araruama I (6.5 to 12.3 μm and 2.4 to 6.5 μm in diameter, respectively) both isolated from a narrowly ocean-connected, organically enriched, hypersaline lagoon [62]; the 4–5.1 μm in diameter *Beggiatoa* sp. MS-81-6, isolated from the marine-influenced Sippewissett Marsh near Woods Hole, Massachusetts, USA [61]; the 3–5 μm in diameter *Beggiatoa* sp. 35Flor from a microbial consortium of a black band disease of an epibenthic scleractinian coral [63]; the uncultured *Beggiatoa* spp. HMW (6–10 μm in diameter) collected from the Hakon Mosby mud volcano in Barents Sea (1,250 m depth) [64]; and *Beggiatoa* sp. MS-81-1c, (1.6–2.2 μm in diameter) also from the marine-influenced Sippewissett Marsh near Woods Hole, Massachusetts, USA. Thus, “*Ca*. V. ishoeyi” shares a similar, macrobacteria morphology and a marine or marine-related habitat with the related organisms within this clade.

It should also be emphasized that “*Ca*. V. ishoeyi” is clearly close to the tree root, which is consistent with the old age, at least Mesozoic, of the HS off Chile [12]. Also, consistent with the consensus criterion to define a new prokaryotic species, less than 97% in identity of the 16S rRNA gene sequences, which corresponds to 70% DNA-DNA hybridization [65], and less than 95% for a new genus [66], “*Ca*. V. ishoeyi” appears as a valid new genus and species within the *Thiotrichaceae* family.

### Nitrate reduction

One of the main features of marine sulfur-oxidizing, vacuolate, megabacteria (larger than *ca*. 10 μm in diameter), is their ability to store high concentrations of nitrate in large vacuoles, as the terminal electron acceptor for the oxidation of sulfide [29]. This is the case for *Thioploca araucae* [67, 68], (“*Ca*. Marithioploca araucae”) [25, 26], and *Thioploca chileae* [67] (“*Ca*. Marithioploca chileae”) [25, 26] of the HS; *“Ca*. Isobeggiatoa divolgata” and “*Ca*. Parabeggiatoa communis” from Limfjorden (Denmark) [30]; “*Ca*. Maribeggiatoa vulgaris” from Guaymas Basin [31, 32]; and “*Beggiatoa* sp.” (“*Ca*. Maribeggiatoa”) from Monterey Canyon [69, 70]. Nevertheless, also non-vacuolate sulfur-oxidizing giant filamentous bacteria use nitrate as a terminal electron acceptor [15, 16].

**Fig 5:**
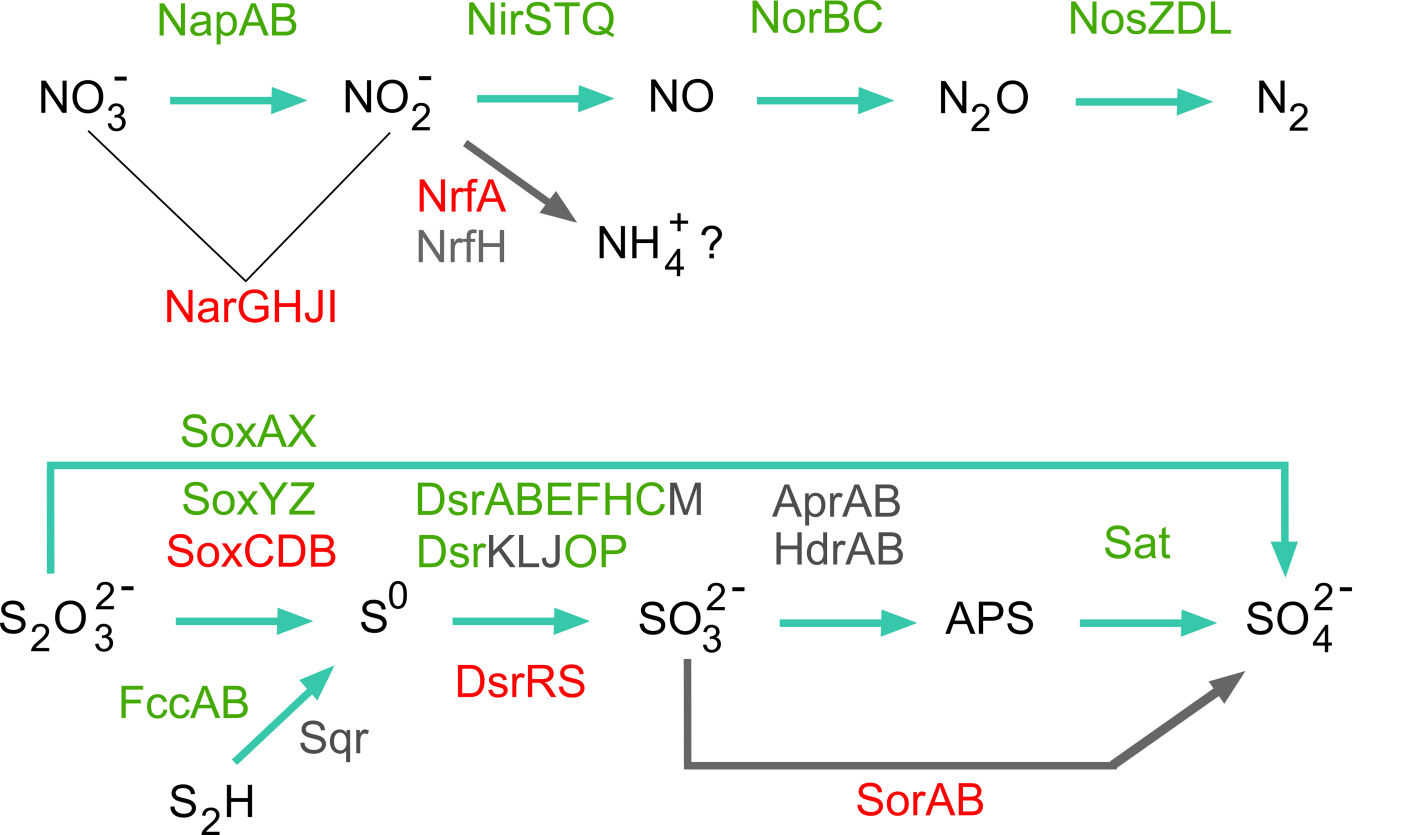
Dissimilatory nitrate reduction, ammonification, and sulfur oxidation. Predicted proteins identified in the draft genome of “*Ca*. V. ishoeyi” are shown in green, identification of close homologue proteins are shown in grey, and proteins identified in other related taxa, but not in “*Ca*. V. ishoeyi”, are shown in red.

Gene prediction for “*Ca*. V. ishoeyi” indicates the presence of a dissimilatory nitrate reduction (denitrification) pathway leading to N_2_ as final product, and likely ammonification, which is the dissimilatory reduction of nitrate to ammonia (DNRA) (Fig 5). The first step into denitrification is conducted by the respiratory nitrate reductase (NAR) and also the periplasmic nitrate reductase (NAP) enzymes, while NAP can as well acts in DNRA [71]. In the genome of “*Ca*. V. ishoeyi”, *napAB* genes (MBHS_00506 and MBHS_00509), encoding for a periplasmic nitrate reductase were identified. On the contrary, *narGHJI* genes encoding for NAR enzymes were not identified in “*Ca*. V. ishoeyi”. The same result was obtained with the macrobacteria *Beggiatoa alba* B18LD (BioProject PRJNA224116), which only harbors NAP enzyme encoding genes. Conversely, in megabacteria “*Ca*. Maribbegiatoa sp.” [31, 32], “*Ca*. Isobeggiatoa divolgata” [30], “*Ca*. Thiomargarita nelsonii Bud S10” [35], and in “*Ca*. Thiomargarita nelsonii Thio36” [34], NAR and NAP encoding genes have been identified. NAP is expressed under aerobic and anaerobic conditions [72], but its role is not totally clear, since it has been found predominantly associated to aerobic denitrification [72]. Moreover, in nitrate ammonifiers NAP is highly effective under low nitrate conditions and its effectiveness is even higher when a reduced carbon source is available for bacterial growth [71]. However, Li *et al*. (2012) [73] reported that in *Magnetospirillum gryphiswaldense* NAP is required for anaerobic growth instead of NAR. On the other hand, Mußmann *et al*. (2007) [30] state that NapAB could allow a large vacuolate filamentous *Beggiatoa*, to respire nitrate at low nitrate concentrations, or even under aerobic conditions.

Two copies of the *nirS* gene (MBHS_03552–03560), encoding for nitrite reductase precursors, were identified close to each other in scaffold scf_477. In a contiguous position, both the *nirT* (MBHS_03559) and the *nirQ* (MBHS_03566) genes were identified that encode for regulatory components. The nitrite removal from the cell is essential, since this compound is generally toxic to bacteria, thus, if the process of nitrite diffusing through the membrane is not efficient, it becomes necessary to convert nitrite to a less toxic form such as N_2_O (nitrous oxide), N_2_ (dinitrogen), or NH_4_^+^ (ammonium). It should be noted that the gene encoding for the pentaheme catalytic subunit (NrfA), involved in dissimilatory nitrite reduction to ammonium [74] was not identified in “*Ca*. V. ishoeyi”. However, the presence of an nrfH homologously encoding a subunit of nitrite reductase complex (MBHS_04162), which mediates the electron transfer from the quinol to the catalytic subunit (NrfA), was identified. The NrfH protein has been originally described in the anaerobic epsilon-proteobacterium *Wolinella succinogenes*, predicted to be a membrane-bound tetrahaem cytochrome c belonging to the NapC/NirT family [75]. Thus, the presence of genes encoding for the enzymes NrfH and NapAB suggests that “*Ca*. V. ishoeyi” could obtain energy through nitrate reduction to ammonia, besides denitrification. Both these capacities are of interest with respect to understanding fundamental regional oceanographic processes, in particular among the traditionally high productivity regions, such as the Humboldt Current ecosystem and the Benguela Current ecosystem, where sulfur oxidizing bacteria are present today, but may have been even more dominant in the geological past [76]. Similar ways for nitrite reduction have been reported for “*Ca*. Maribeggiatoa sp.” [32] and “*Ca*. Isobeggiatoa divolgata” [30], which, besides both NAR and NAP enzymes present a candidate gene that would encode for an octaheme cytochrome nitrite reductase capable for DNRA. On the other hand, “*Ca*. Thiomargarita nelsonii Thio36” [34], “*Ca*. Thiomargarita nelsonii Bud S10” [35], *Thioploca ingrica* [33], and *Beggiatoa alba* B18LD, contain genes encoding for the assimilatory nitrate reductase (*nasA*) and nitrite reductase (*nirBD*) enzymes, involved in both assimilatory and dissimilatory pathways. Conversely, *nasA* nor *nirBD* genes, were identified in the draft genome of “*Ca*. V. ishoeyi”.

The third intermediate in the denitrification pathway is nitrous oxide (N_2_O), generated by the nitric oxide reductases. In the genome of “*Ca*. V. ishoeyi”, the *norC* gene (MBHS_03562), encoding for nitric oxide reductase subunit C and the *norB* gene (MBHS_03563), encoding to nitric oxide reductase subunit B, were identified in a contiguous segment of the genome.

The last stage of denitrification is the nitrous oxide reduction generating N_2_ as final product. The gene cluster involved in the direct production of molecular nitrogen including *nosZ* gene (Nitrous-oxide reductase precursor) (MBHS_04541, splitted into two segments), *nosD* gene (MBHS_04547) (Periplasmic copper-binding protein), and the *nosL* gene (MBHS_04551) (accessory protein of *nos*), were identified in “*Ca* V. ishoeyi”. Among related genomes, the *nosZ* gene has been previously found only in *Thioploca ingrica* [33], “*Ca*. Thiomargarita nelsonii Bud S10” [35], and “*Ca*. Thiomargarita nelsonii Thio36” [34].

Evidence suggests that “*Ca*. V. ishoeyi” would be capable of respiring nitrate through both pathways: denitrification and DNRA. These alternatives give “*Ca*. V. ishoeyi” the metabolic versatility needed by an organism facing the challenges arising in a very dynamic and variable environment, including a great variation in redox conditions, even in the microscale. Therefore, it is highly probable that “*Ca*. V. ishoeyi” can comfortably occupy the sediment surface, but also could sustain anoxic periods and/or occupy deeper anoxic sediment conditions.

### Sulfur oxidation

Regarding the fact that the HS is a highly sulfidic environment and intracellular sulfur globules could be observed in “*Ca*. V. ishoeyi” filaments under the microscope, adaptation to sulfide oxidation could be expected. Accordingly, we were able to identify most of the genes (S2 Table) required for the oxidation of sulfide, sulfur and thiosulfate to sulfate, shared by many free-living Gammaproteobacteria such as; *Thiomicrospira*, *Halothiobacillus*, and *Beggiatoa* [77–79].

Sulfur bacteria separate the two half reactions and oxidize sulfide to elemental sulfur using nitrate when present in lower, sulfidic layers of the sediment, after which filaments move up to the oxic zone where the stored elemental sulfur is oxidized to sulfate using oxygen [20, 30].

**Fig 6:**
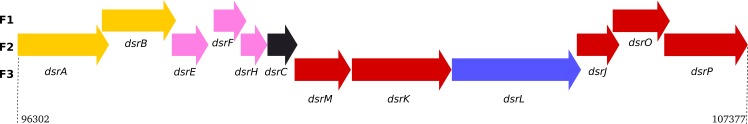
Schematic representation of the dissimilatory sulfite reductase (DSR) system, identified in “*Ca* V. ishoeyi”. In yellow: *dsrAB* genes coding for sulfite reductases: in pink: *dsrEFH* genes, coding for heterohexameric protein; in black: the *dsrC* gene, coding for sulfurtransferase; in red: *dsrMKJOP* genes, coding for transmembrane electron-transporting complex; and, in blue: the *dsrL* gene, coding for intracellular sulfur oxidation protein, are shown. F1, F2, and F3 represent the frame arrangement in the sense strand DNA.

“*Ca*. V. ishoeyi” could carry out sulfide oxidation through the flavocytochrome c/sulfide dehydrogenase encoded by *fccAB* genes (Fig 5). Furthermore, a close homologue of the *sqr* gene sequence, encoding the other sulfide oxidation enzyme sulfide-quinone oxido-reductase (MBHS_00764), was identified. Apparently, in “*Ca*. V. ishoeyi” the oxidation of elemental sulfur to sulfite is carried out through the dissimilatory sulfite reductase (DSR) system (Fig 6). The sulfite reductase encoded by *dsrAB* genes (MBHS_03945–03946), the soluble cytoplasmic protein (DsrC: Sulfurtransferase), encoded by the *dsrC* gene (MBHS_03950, and the transmembrane electron-transporting complex, encoded by *dsrMKJOP* genes (MBHS_03951-03952-03954-03955-03956), were identified in a contiguous position. Next, in an adjacent position, the heterohexameric protein, encoded by the *dsrEFH* genes (homologous to the TusBCD complex) (MBHS_03947-03948-03949), an effective sulfur donor for DsrC in *Allochromatium vinosum*, was identified [80]. Also, a close homologue to *Thioploca ingrica*’s intracellular sulfur oxidation protein, DsrL (MBHS_03953), was identified. All ORF candidates for the DSR system were identified in a contiguous segment.

The prediction for *fccAB* genes and a homolog of the *sqr* gene in “*Ca*. V. ishoeyi” would allow regulating the sulfide oxidation, depending on the environment conditions. It is reasonable to hypothesize that “*Ca*. V. ishoeyi” has been shaped by the evolution and selection to thrive in the upper microoxic benthic layers, under oxygen deficient water masses such as those of the Peru-Chile Subsurface Countercurrent [24].

A similar sulfide oxidation method has been reported in close bacterial genomes, *i*.*e*., “*Ca*. Isobeggiatoa divolgata”, “*Ca*. Parabeggiatoa communis” [30], “*Ca*. Maribeggiatoa vulgaris” [32] *Beggiatoa leptomitoformis* D-402^T^ [81], *Beggiatoa alba* B18LD [34, 81], and *Thioploca ingrica* [33]. Gene sequences encoding for AprAB enzymes, adenosine-5’-phosphosulfate reductase (MBHS_04595–04596), HdrAB; heterodisulfide reductase (MBHS_00323–00322); and the sequence of the *sat* gene (MBHS_00245) encoding for sulfate adenylyltransferase, which is used to oxidize sulfite to sulfate, were identified in “*Ca*. V. ishoeyi”. The HdrAB are featured as a flavo-FeS protein [82], which together with HdrC form a soluble complex with the F_420_ -non-reducing hydrogenase that catalyzes reduction of the heterodisulfide by hydrogen [83]. However, genes encoding the protein HdrC or a homologue were not identified in “*Ca*. V. ishoeyi”; neither could we identify *sor* genes, which would code an enzyme that directly mediates sulfite oxidation to sulfate.

“*Ca*. V. ishoeyi” appears to be capable of thiosulfate oxidation by the Sox (sulfur oxidation) system which consists of three core parts with SoxAX, SoxYZ, and SoxB [84–86], and which are suggested essential to thiosulfate oxidation in this system [33, 87]. Two copies of the gene coding for SoxAX (MBHS_04050 and MBHS_04079) were identified in two contiguous scaffolds, adjacent to the genes encoding for SoxY and SoxZ (MBHS_04077–04078) proteins. However, the SoxB encoding genes or homologous sequences were not identified, perhaps due to assembly errors in the draft genome, or lack of sequence coverage to assemble into a contig of >1000 bp. Moreover, in “*Ca*. V. ishoeyi” the SoxCD encoding genes or homologous sequences were not identified, which is accordance with reported observations that sulfur-oxidizing bacteria typically lacks the SoxCD genes [88, 89], when the DSR system genes is present, except in environmental fosmid sequences [33].

Our results from the genome analysis confirm that “*Ca*. V. ishoeyi” can obtain energy from sulfide and sulfur oxidation, maintaining a chemolithoautotrophic mode of life, where its intracellular sulfur globules would function as a stored energy source.

### Oxygen and dimethyl sulfoxide respiration

The draft genome of “*Ca*. V. ishoeyi” encodes the high-affinity Cbb3-type cytochrome c oxidase subunit CcoP2 (MBHS_02490). The Cbb3-type cytochrome c oxidase is activated under microoxic conditions to oxidize inorganic sulfur species [30, 90]. Thus, the presence of the subunit CcoP2 from the Cbb3-type cytochrome c oxidase suggests that “*Ca*. V. ishoeyi” would be able to use oxygen as terminal electron acceptor under microoxic conditions to growth. Conversely, the low-affinity cytochrome c aa3-oxidase, is activate under high oxygen conditions [30], was not identified in “*Ca*. V. ishoeyi”. Additionally, “*Ca*. V. ishoeyi” could respire dimethyl sulfoxide (DMSO), due to the presence of the *dsmA* (MBHS_03149) and *dsmB* (MBHS_03148) genes, which encode for DMSO reductases. The DMSO reduction represents an alternative respiration pathway, since (CH_3_)_2_OS is found in significant concentrations in aquatic environments, sometimes representing the most abundant methylated sulfur compound present [91].

In *Thioploca ingrica* a complete ccoNOQP gene cluster, encoding for the high-affinity cytochrome c bb3-oxidase, as well as the DMSO reductases, have been identified, but no genes encoding for the low-affinity cytochrome c aa3-oxidase [33]. In addition, the recently published genome of “*Ca*. Thiomargarita nelsonii Thio36” [34] harbors genes encoding for high- and low-affinity cytochrome c oxidases, as well the *dmsA* gene. Besides, in “*Ca*. Isobeggiatoa divolgata” and “*Ca*. Parabeggiatoa communis”, both high- and low-affinity terminal oxidases, plus *dmsABC* genes were identified [30]. Thus, results indicate that “*Ca*. V. ishoeyi”, as several other sulfur-oxidizing macrobacteria have a flexible response when facing changes in oxygen concentration, being able to use different respiration pathways. This plasticity in respiratory pathways would allow “*Ca*. V. ishoeyi” inhabiting the variable oceanographic conditions over the HS [92].

### Carbon metabolism

Most of currently available *Thiotrichaceae* genomes harbor protein-coding genes for the tricarboxylic acid cycle (TCA cycle) and the genome of “*Ca*. V. ishoeyi” is no exception, harboring genes coding for almost the full set of proteins that constitute the TCA cycle (S3A Table). Among them, we found the *sucA* (MBHS_00167) and *sucB* genes (MBHS_01480–04108), encoding for the 2-oxoglutarate dehydrogenase complex, an essential enzyme in the TCA cycle. The absence of this enzyme has been indicated as a key feature for obligate autotrophy [93], thus its presence would be characteristic of an organism living under conditions of variable amounts of sulfur that could take advantage of a variety of organic compounds, such as carbon-containing molecules or other energy sources, as a facultative chemolithoautotrophic. Moreover, according to earlier studies on the marine non-vacuolate *Beggiatoa*, the ability to utilize a wide range of organic compounds was described [94]. On the other hand, the presence of the complete protein-coding genes for the TCA cycle has been reported in other related genomes *i*.*e*., *Thioploca ingrica*, *Beggiatoa* sp. 35Flor, and “*Ca*. Thiomargarita nelsonii Thio36” [34].

It should be noted that in “*Ca*. V. ishoeyi” most of the genes required to encode the glycolysis pathway were identified (S3B Table). However, “*Ca*. V. ishoeyi” lacks the *pdhAB* genes, coding for pyruvate dehydrogenase; the *pdhC* gene, coding for dihydrolipoamide acetyltransferase, and the ppgk gene, coding for polyphosphate glucokinase. A similar condition has been reported for *Beggiatoa* sp. 35Flor [95]. The lack of these genes could be a feature of the non-vacuolated sulfur-oxidizing bacteria. However, additional genomic studies are required to discard the possibility that absence of these genes is due to incomplete assembly.

The *ccbM* gene (MBHS_00487) coding for form II of the ribulose-bisphosphate carboxylase oxygenase (RubisCO), which mediates CO_2_ fixation, was identified in “*Ca*. V. ishoeyi”. Besides this finding, five other genes coding for enzymes of the Calvin–Benson–Bassham cycle (S3C Table), were identified in “*Ca*. V. ishoeyi”, but the gene coding for form I RuBisCO was not found. On the other hand, *aceA* and *aceB* genes, coding for key enzymes in the glyoxylate cycle: isocitrate lyase and malate synthase, were not identified in “*Ca*. V. ishoeyi”. The glyoxylate cycle can bypass the TCA, to form glyoxylate and succinate from isocitrate for gluconeogenesis, using compounds such as acetate. The full set of genes coding for enzymes that participate in the glyoxylate cycle has been identified in the genome of *Beggiatoa* sp. 35Flor, *Beggiatoa alba* B18LD, and *Thioploca ingrica*.

The protein-coding gene for acetate assimilation, a*ctP* (MBHS_02529), which encodes for a cation/acetate symporter, and two copies of the *acsA* gene (MBHS_00422–03461), encoding for an acetyl-CoA synthetase, which mediates the transformation from acetate to acetyl-CoA, were, identified in “*Ca*. V. ishoeyi”.

These findings suggest that “*Ca*. V. ishoeyi” could behave as a facultative chemolithoautotroph, being able to use acetate as an alternative energy source, and CO_2_ fixation, using inorganic carbon source from sediments, such as bicarbonate to form biomass.

### Phosphorus metabolism

The ability of *Beggiatoa* to store phosphorus as polyphosphate has been experimentally documented for the narrow non-vacuolated *Beggiatoa* sp. strain 35Flor [18, 19], and was further demonstrated in natural samples of large vacuolated megabacteria [17–19]. Therefore, natural communities of macro- and megabacteria can play an important role in removing phosphorus from the marine environment and releasing it back in, a feature already proposed earlier following the observation of extensive *Thioploca* mats and the abundance of large phosphorite deposits off western South America [76, 96]. Moreover, these findings, in combination with reported oceanographic conditions prevailing in the Proto-Humboldt Sulfuretum could add a new potential explanation for the unusual phosphatic preservation of Oxfordian Jurassic marine life as found in the “Cordillera Domeyko”, in northern Chile [12].

According to the annotation, 20 CDSs related to the phosphorus metabolism were identified in the genome of “*Ca*. V. ishoeyi”, among them, genes encoding for the transport, biosynthesis, and degradation of polyphosphates. The *pitA* gene (MBHS_01834), identified in “*Ca*. V. ishoeyi” coding for a phosphate inorganic transport (Pit), is indicated as a low-affinity transporter taking up inorganic phosphate through the phosphate transport system. However, the high-affinity transporter (*pstSBCA*) was not identified. Moreover, the polyphosphate kinase (PPK2) encoding gene (MBHS_02186) was identified in the scf_436 scaffold. The synthesis of polyphosphates in prokaryotes is mediated mostly by PPK1, which was not identified in “*Ca*. V. ishoeyi”, and PPK2 which prefers ATP rather than GTP (guanosine-5'-triphosphate), and Mg^2+^ over Manganese. PPK2 has also been found in *Pseudomonas aeruginosa* as an enzyme catalyzing GTP synthesis from GDP and polyphosphate. Conversely to PPK1, PPK2 preferentially catalyzes the reverse reaction [97]. Once the polyphosphate is synthesized, it may be stored intracellularly and generate phosphate from the degradation of polyphosphate by exopolyphosphatase (PPX), which catalyzes the hydrolysis of terminal phosphate residues from long chain polyphosphate [98]. Then, the phosphate may be transported out of the cell by the Pit transport system. The *ppx* gene (MBHS_00946) was identified in “*Ca*. V. ishoeyi”, which encodes for PPX. In other sulphur-oxidizers such as *Thioploca ingrica*, *ppk* and *ppgk* genes, encoding for polyphosphate glucokinase have been identified [33]. *Ppgk* catalyses the phosphorylation of glucose and glucosamine to glucose-6-phosphate and glucosamine-6-phosphate respectively, however, it was not identified in “*Ca*. V. ishoeyi”. Also, *ppgk* has been reported in “*Ca*. Isobeggiatoa” and “*Ca*. Parabeggiatoa” from Eckernförde Bay, Germany, Baltic Sea [30]. Furthermore, under laboratory conditions, Brock *et al*. (2012) [19] confirm the presence of polyphosphate inclusions in *Beggiatoa* sp. 35Flor which are enclosed by a lipid layer and store cations. The same authors further suggest that these inclusions “represent a new type of membrane-surrounded storage compartment within the genus *Beggiatoa*, distinct from the mostly nitrate-storing vacuoles known from other marine sulfur-oxidizing *Thiotrichaceae*”.

The presence of genes related to transport, biosynthesis, and degradation of polyphosphates suggests that “*Ca*. V. ishoeyi” would be capable of storing polyphosphates, however, experimental testing including the visualization of potential phosphate inclusions as suggested by Brock *et al*. (2012) [19] would be necessary to confirm this.

### CRISPR–Cas system

The CRISPR–Cas (clustered regularly interspaced short palindromic repeats–CRISPR-associated proteins) modules are adaptive immunity systems that are encoded by most Archaea and many Bacteria to act against invading genetic elements such as bacteriophages and plasmids [99, 100].

Through the CRISPRFinder tool [51], 18 CRISPR repeats or Direct Repeats (DRs) were identified in “*Ca*. V. ishoeyi”. These consist of a succession of highly conserved regions (S4A Table), which varied between 26 and 35 bp. Remarkable is the CRISPR repeat DR-16 (“GTTGACTGCCGCACAGGCAGCTTAGAAA”) consists of 28 bp and 110 spacers, which covers a size of 6,750 bp in the scf_483. A total of 33 CRISPR-associated (Cas) protein encoding genes, were detected in *“Ca*. V. ishoeyi” (S4B Table), most of them, associated with a DR. Cas proteins are directly implicated in different stages of the processing of CRISPR loci transcripts, *i*.*e*., cleavage of the target DNA or RNA and new spacer integrations which correspond to alien DNA fragments that are included in the CRISPR cassettes [100, 101]. Thus, in “*Ca*. V. ishoeyi” genes encoding for proteins such as: Cas4/endonuclease, Cas1 fusion (MBHS_00983), non-defined Cas; and RAMP Superfamily protein (MBHS_00987-00989-00990-009929, and Cmr5 protein (MBHS_00991) were identified associated to DR-4. Moreover, flanking to the DR-8 CRISPR repeat, a noteworthy gene array, coding for an enzymatic complex of CRISPR-Cas, was identified (9 spacers). Here, genes coding for the key protein Cas1 (MBHS_01576) that allows the CRISPR system memorize previous contacts with infectious agents [101], and *cas3* (MBHS_01575), coding for CRISPR-associated nuclease/helicase Cas3, which acts as a single-strand nuclease and helicase in the CRISPR-Cas immune system [102], together with the protein-encoding genes *cys1*, *cys2*, *cys3*, and *cas6f* (MBHS_01573-01572-01571-01570), were identified. This gene array conforms the canonical type I-F *cas*, which encode for a I-F CRISPR-Cas system. The type I-F CRISPR-Cas system has been identified and well characterized in bacteria such as *Pseudomonas aeruginosa* [103], where it has been demonstrated that under lab conditions it is capable of acquiring protective spacers during challenges by the bacteriophage DMS3*vir* [104].

Noticeable is the finding that the set of Cas protein-encoding genes identified in “*Ca*. V. ishoeyi” is significantly larger than those found in other related genomes, *e*.*g*., *Thioploca ingrica*, with seven genes: *cas4*, *cas2*, *cas1*, *cas5*, *cas3*, *csh2*, and TM1802 [33]; “*Ca*. Isobeggiatoa” with three genes: TM1793, *cas3*, and TM1801 [30]. In *Beggiatoa alba*, six repeat regions and two genes encoding for *cas1* and RAMP Superfamily protein (BioProject PRJNA224116), were identified, while in *Beggiatoa leptomitiformis* [37], and “*Ca*. Parabeggiatoa” [30] no Cas protein-encoding genes have been identified.

Indeed, the numerous CRISPR repeats detected could well reflect the pressure that bacteriophages have exerted on these organisms along the geological time scale. Morphologically similar to “*Ca*. V. ishoeyi” fossil prokaryotic filamentous forms have been cited between *ca*. 3,496 and 2,560 million years ago within the Archean Eon by Schopf [8]. These findings have given rise to a whole system of generic names such as: *Archeoscillatoriopsis* sp. (4–16 μm in diameter), and binomial designations such as *Archaescillariopsis disciformis* (*ca*. 3–5 μm in diameter). Significantly, according to Schopf’s compilation work, broad tubular and sheath-like structures first appeared 2,560 and 2,516 million years ago and were named *Siphonophycus transvaalense* (*ca*. 10–28 and 15–27 μm in diameter), perhaps signaling the advent of oxygen at the bottom and the setting of a selection process that lead to a vacuolate, *Thioploca*-like morphology.

The presence in “*Ca*. V. ishoeyi” of canonical type I-F cas genes, together with eighteen CRISPR repeats suggests with high probability that “*Ca*. V. ishoeyi” is capable of generate a strong defense against bacteriophages. In addition, considering the proximity of “*Ca*. V. ishoeyi” to the root of the phylogenetic tree and the old age of sulfuretum ecosystems on Earth, we here tentatively posit that, besides being a good taxonomic discriminator and a measure of the actual bacteriophage pressure, the presence and complexity of the CRISPR-Cas system could be a good measure of the species age and the ecological maturity of a given prokaryotic community, with advantages for taxonomy and nomenclature.

### Secondary metabolites

Although not thoroughly investigated in sulfur-oxidizing bacteria, several enzyme-encoding gene clusters involved in the biosynthesis of secondary metabolites have been identified, *i*.*e*., non-ribosomal peptide synthetase (NRPSs), and polyketide synthetase (PKSs) genes [30], presumably originating from cyanobacteria, as suggested after genome analysis of other vacuolated sulfur bacteria [30, 31]. For example, a new macrolide compound, denominated Macplocimine A, was isolated from massive *Thioploca*-dominated mat samples, collected off central Chile [105].

**Fig 7:**
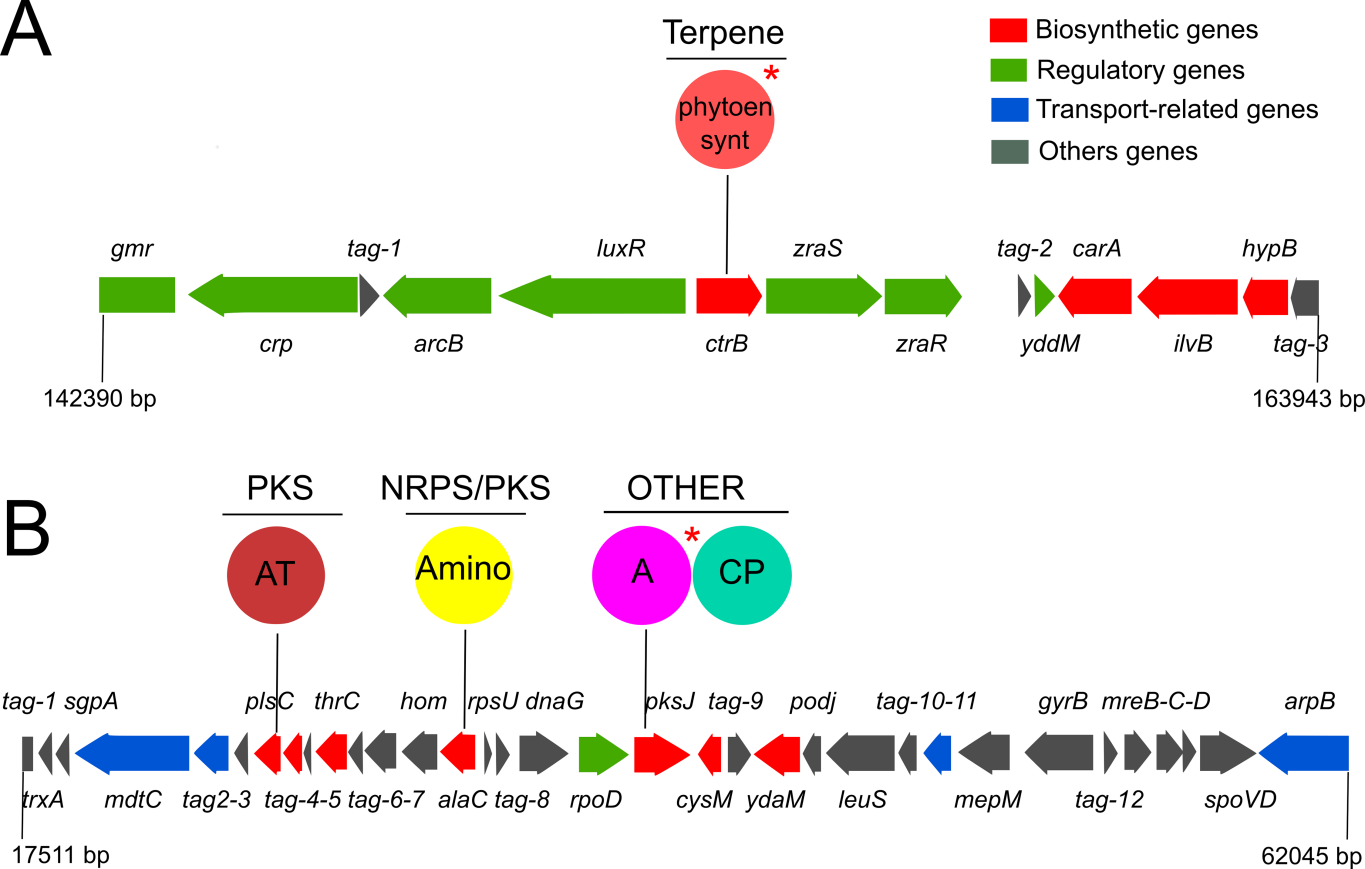
Biosynthetic gene clusters, identified as terpene and undetermined type in “*Ca*. V. ishoeyi”. **A. Terpene gene cluster.** Sphere indicates the Phytoene synthase (phythoen_synt) domain. **B**. **Undetermined biosynthetic gene cluster.** Spheres show the Acyltransferase (AT), involved in biosynthesis of Polyketide synthase (PKS) compounds, Aminotransferase (Amino), involved in biosynthesis of Polyketide synthase/nonribosomal peptide synthetases (PKS/NRPS) compounds, Adenylation (A) and Carrier Protein (CP; Thiolation) domains, from undetermined compounds (Other). Asterisks indicate domains used as a rule for gene cluster classification. Identified genes are indicated in the backbone of the gene clusters and the product list is available in the S5A and S5B Table.

In “*Ca*. V. ishoeyi”, the presence of gene clusters related to the biosynthesis of secondary metabolites was assessed through the antiSMASH tool [47–49]. A gene cluster of 21,082 bp containing genes for the synthesis of a terpene type compound, which includes a wide range of complex natural products such as toxins, repellents, and attractants [106], and an undetermined gene cluster of 41,958 bp, were identified in “*Ca*. V. ishoeyi”. The terpene gene cluster candidate (Fig 7A) contains a key gene sequence coding for a phytoene synthase (MBHS_04759). On the other hand, the undetermined gene cluster has a sequence identified as *pksj* gene (MBHS_04255), encoding Adenylation (A) and Carrier Protein (Thiolation) domains, similar to a non-ribosomal peptide-synthetase (NRPS) module. However, it lacks the condensation (C) domain to complete a typical NRPS module (Fig 7B). Therefore, it is highly probable that the enzyme encoded by the *pksj* gene is involved in the biosynthesis of some non-canonical secondary metabolite, *e*.*g*., in non-proteinogenic amino acid biosynthesis, such as many enzymes with A-CP domain structures that are not ‘real’ NRPSs (Personal communication from Dr. Marnix Medema, Wageningen University, Netherlands).

The phylogenetic analysis using the protein sequence of phytoene synthase detected in “*Ca*. V. ishoeyi” (S2 Fig), shows that “*Ca*. V. ishoeyi” is located between the root of the tree (*Acidithiobacillus caldus*—WP_004873) and the monophyletic clade composed by *Thioploca ingrica* (WP_045476987), *Beggiatoa* sp. IS2 (WP_045476987), *Beggiatoa leptomitiformis* (WP_062151885), “*Ca*. Thiomargarita nelsonii” (KHD05642), “*Ca*. Marithrix” Canyon 246 (WP_069469840), and *Beggiatoa* sp. PS (EDN68199). Moreover, the protein sequence of phytoene synthase detected in “*Ca*. V. ishoeyi" and the sequences from the bacteria belonging to the contiguous clade, share a coverage and identity that ranging between 92%-93% and 57%-60%, respectively.

About 50,000 terpenoid metabolites within nearly 400 different structural families are known, mostly isolated from plants and only a few originating from prokaryotes. However, in recent studies, using bacterial proteins from public databases [107], 262 putative bacterial terpene synthases were identified. The phytoene synthase is a key enzyme in biosynthesis of carotenoids, natural pigments synthesized by plants, and some microorganisms that carry out important physiological functions. Thus, they are essential for photosynthesis and photoprotection in photosynthetic organisms, while in non-photosynthetic organisms, they take part in mitigating photooxidative damage [108]. Despite the presence of phytoene synthases in several *Thiotrichaceae*, the possible role in them and in “*Ca*. V. ishoeyi” is not clear since they are neither photosynthetic organisms not are they exposed to sunlight. Therefore, its presence and function is an issue to be clarified.

## Conclusions

The draft genome of a marine, non-vacuolated filamentous macrobacterium, inhabiting strongly reduced soft sediment, in the Humboldt Sulfuretum, off central Chile, was successfully obtained.

Here the new bacterium, “*Candidatus* Venteria ishoeyi” (“*Ca*. V. ishoeyi”), within the family *Thiotrichaceae*, is proposed. The phylogenetic analysis indicated that the “*Ca*. V. ishoeyi” affiliated closely with other marine and hypersaline narrow non-vacuolate *Beggiatoa* spp. close to the phylogenetic root of the family *Thiotrichaceae*. Although “*Ca*. V. ishoeyi” lacks a nitrate-storing vacuole, genes coding for enzymes enabling both, denitrification and possibly also ammonification, were identified, which is a feature that was already detected in some other members of the family of large sulfur bacteria [34, 35]. Besides, genes for sulfur oxidation, oxygen, and dimethyl sulfoxide respiration under micro-oxic conditions, and organic and inorganic carbon fixation, were identified in "*Ca*. V. ishoeyi", suggesting a facultative chemolithoautotrophic lifestyle. Moreover, judging from the possession of a comparatively richly endowed CRISPR-Cas system, it is suggested that within its ancient and competitive habitat, “*Ca*. V. ishoeyi” in time has undergone, and presently is, subjected to great pressure from bacteriophages, within the highly diversified HS filamentous microbial community, which according to the micropaleontology may have already existed for Eons.

## Supporting information

S1 Fig Amplification products of 16S rDNA genes and whole genome amplification (WGA) from seven bacterial filaments (MDA1-7) isolated by micromanipulation and visualized using agarose gel electrophoresis. The top of the figure shows the amplification products of the full length (~1.5 kbp) 16S rDNA PCR products of the seven micro-manipulated bacterial filaments (MDA1—MDA7). The PCR products were purified and sequenced using 27F and 1492R primers by Sanger sequencing and visualized by agarose gel electrophoresis (1%) E-gels (Invitrogen) with a low DNA mass 2kb ladder. The bottom of the figure (MDA-DNA) shows the products of whole genome amplification (WGA) of the single filaments MDA1 to MDA7, and the MDA no template control (NTC) visualized through agarose gel electrophoresis (1%) E-gels (Invitrogen).
(PDF)

S2 Fig Phylogenetic analysis for the amino acid sequence of phytoene synthase identified in “*Ca*. V. ishoeyi”. The aligning of the amino acid sequence of the phytoene synthase (ps) identified in “*Ca*. V. ishoeyi” and 25 amino acid sequences of the Squalene/Phytoene synthase (Sps) and Phytoene synthases taken from the top 50 blastp match, and a sequence of *Acidithiobacillus caldus* (WP_004873208) as root, was used to build the phylogenetic tree.
(TIF)

S1 Table Identification of amplified filaments by SeqMatch function (Ribosomal Database Project, RDPII). (PDF)

S2 Table Genes coding for sulfur oxidation enzymes in “*Ca*. V. ishoeyi”. (PDF)

S3 Table Genes identified in “*Ca*. V. ishoeyi”: S3A. In the tricarboxylic acid cycle; S3B. Involved in glycolysis; S3C. Involved in the Calvin-Benson-Bassham cycle.
(PDF)

S4 Table **S4A. List of CRISPR repeats identified in “*Ca*. V. ishoeyi”.** The table shows: scaffolds containing start and end of CRISPR repeat regions, Direct Repeat (DR) sequences, length of the DR, and number of spacers. **S4B. CRISPR-associated protein encoding genes identified in “*Ca*. V. ishoeyi”**.
(PDF)

S5 Table S5A. Gene and product names of the Terpene type gene cluster. S5B. Gene and product names of the gene cluster, related with the potential biosynthesis of a non-classified secondary metabolite.
(PDF)

## References

[1] Gallardo VA, Espinoza C, editors. Large multicellular filamentous bacteria under the oxygen minimum zone of the eastern South Pacific: a forgotten biosphere Optical Engineering+ Applications; 2007: International Society for Optics and Photonics.

[2] Gallardo VA, Espinoza C. New communities of large filamentous sulfur bacteria in the eastern South Pacific. International Microbiology. 2010;10(2):97–102. 17661287

[3] Gallardo VA, Espinoza C, Fonseca A, Musleh S. Las grandes bacterias del Sulfureto de Humboldt. Gayana (Concepción). 2013;77(2):136–70.

[4] Gallardo V, Fonseca A, Musleh S, Espinoza C. Extrapolations of Standing-Stocks of Big Bacteria in Humboldt Eastern Boundary Current Ecosystem (HEBCE). Oceanography: Open Access. 2013;2013.

[5] Gallardo VA, Fonseca A, Espinoza C, Ruiz-Tagle N, Musleh S. Bacteria of the Humboldt sulfuretum comply with unifying macroecological principles. Marine Biodiversity. 2015:1–8.

[6] Baas-Becking L. Studies on the sulphur bacteria. Annals of Botany. 1925;39(155):613–50.

[7] Tremberger G Jr, Gallardo V, Espinoza C, Holden T, Gadura N, Cheung E, et al, editors. Archaeon and archaeal virus diversity classification via sequence entropy and fractal dimension SPIE Optical Engineering+ Applications; 2010: International Society for Optics and Photonics.

[8] Schopf JW. Fossil evidence of Archaean life. Philosophical Transactions of the Royal Society of London B: Biological Sciences. 2006;361(1470):869–85. doi: 10.1098/rstb.2006.1834 16754604 10.1098/rstb.2006.1834 PMC1578735

[9] Wacey D, Kilburn MR, Saunders M, Cliff J, Brasier MD. Microfossils of sulphur-metabolizing cells in 3.4-billion-year-old rocks of Western Australia. Nature Geoscience. 2011;4(10):698–702.

[10] Schopf JW, Kudryavtsev AB, Walter MR, Van Kranendonk MJ, Williford KH, Kozdon R, et al Sulfur-cycling fossil bacteria from the 1.8-Ga Duck Creek Formation provide promising evidence of evolution's null hypothesis. Proceedings of the National Academy of Sciences. 2015;112(7):2087–92. 10.1073/pnas.1419241112 PMC4343172 25646436

[11] Della Pierre F, Clari P, Natalicchio M, Ferrando S, Giustetto R, Lozar F, et al Flocculent layers and bacterial mats in the mudstone interbeds of the Primary Lower Gypsum unit (Tertiary Piedmont basin, NW Italy): Archives of palaeoenvironmental changes during the Messinian salinity crisis. Marine Geology. 2014;355:71–87.

[12] Schultze H-P. Three-dimensional muscle preservation in Jurassic fishes of Chile. Andean Geology. 1989;16(2):183–215.

[13] Danovaro R, Canals M, Tangherlini M, Dell’Anno A, Gambi C, Lastras G, et al A submarine volcanic eruption leads to a novel microbial habitat. Nature Ecology & Evolution. 2017;1:0144. 10.1038/s41559-017-0144 28812643

[14] Nelson DC, Jørgensen BB, Revsbech NP. Growth pattern and yield of a chemoautotrophic Beggiatoa sp. in oxygen-sulfide microgradients. Applied and Environmental Microbiology. 1986;52(2):225–33. 16347121 10.1128/aem.52.2.225-233.1986 PMC203507

[15] Sweerts J-PR, De Beer D, Nielsen LP, Verdouw H, Van den Heuvel JC, Cohen Y, et al Denitrification by sulphur oxidizing Beggiatoa spp. mats on freshwater sediments. Nature. 1990;344(6268):762–3.

[16] Kamp A, Stief P, Schulz-Vogt HN. Anaerobic sulfide oxidation with nitrate by a freshwater Beggiatoa enrichment culture. Applied and environmental microbiology. 2006;72(7):4755–60. doi: 10.1128/AEM.00163-06 16820468 10.1128/AEM.00163-06 PMC1489373

[17] Schulz HN, Schulz HD. Large sulfur bacteria and the formation of phosphorite. Science. 2005;307(5708):416–8. doi: 10.1126/science.1103096 15662012 10.1126/science.1103096

[18] Brock J, Schulz-Vogt HN. Sulfide induces phosphate release from polyphosphate in cultures of a marine Beggiatoa strain. The ISME journal. 2011;5(3):497–506. doi: 10.1038/ismej.2010.135 20827290 10.1038/ismej.2010.135 PMC3105714

[19] Brock J, Rhiel E, Beutler M, Salman V, Schulz-Vogt HN. Unusual polyphosphate inclusions observed in a marine Beggiatoa strain. Antonie van Leeuwenhoek. 2012;101(2):347–57. doi: 10.1007/s10482-011-9640-8 21909788 10.1007/s10482-011-9640-8 PMC3261416

[20] Fossing H, Gallardoi V, Jørgensen BB, Hüttel M, Nielsen LP, Schulz H, et al Concentration and ttransport of nitrate by the mat-forming sulphur bacterium Thioploca. Nature. 1995;374:20.

[21] Otte S, Kuenen JG, Nielsen LP, Paerl HW, Zopfi J, Schulz HN, et al Nitrogen, Carbon, and Sulfur Metabolism in NaturalThioploca Samples. Applied and Environmental Microbiology. 1999;65(7):3148–57. 10388716 10.1128/aem.65.7.3148-3157.1999 PMC91469

[22] Zopfi J, Böttcher ME, Jørgensen BB. Biogeochemistry of sulfur and iron in Thioploca-colonized surface sediments in the upwelling area off central Chile. Geochimica et Cosmochimica Acta. 2008;72(3):827–43.

[23] Høgslund S, Revsbech NP, Kuenen JG, Jørgensen BB, Gallardo VA, van de Vossenberg J, et al Physiology and behaviour of marine Thioploca. The ISME journal. 2009;3(6):647–57. doi: 10.1038/ismej.2009.17 19262616 10.1038/ismej.2009.17

[24] Gallardo VA. Large benthic microbial communities in sulphide biota under Peru–Chile subsurface countercurrent. Nature. 1977;268:331–2.

[25] Salman V, Amann R, Girnth A-C, Polerecky L, Bailey JV, Høgslund S, et al A single-cell sequencing approach to the classification of large, vacuolated sulfur bacteria. Systematic and Applied Microbiology. 2011;34(4):243–59. doi: 10.1016/j.syapm.2011.02.001 21498017 10.1016/j.syapm.2011.02.001

[26] Teske A, Salman V. The family Beggiatoaceae. The Prokaryotes: Springer; 2014 p. 93–134.

[27] Garrity GM, Bell JA, Lilburn T. Thiotrichalesord. nov. Bergey’s Manual® of Systematic Bacteriology. 2005:131–210.

[28] Rabenhorst. Flora Europaea Algarum aquae dulcis et submarinae: Leipzig, Section II; 1865. 319 p.

[29] Schulz HN, Jørgensen BB. Big bacteria. Annual Reviews in Microbiology. 2001;55(1):105–37. 10.1146/annurev.micro.55.1.105 11544351

[30] Mußmann M, Hu FZ, Richter M, de Beer D, Preisler A, Jørgensen BB, et al Insights into the genome of large sulfur bacteria revealed by analysis of single filaments. PLoS Biol. 2007;5(9):e230 doi: 10.1371/journal.pbio.0050230 17760503 10.1371/journal.pbio.0050230 PMC1951784

[31] MacGregor BJ, Biddle JF, Teske A. Mobile elements in a single-filament orange Guaymas Basin Beggiatoa ("*Candidatus* Maribeggiatoa") sp. draft genome: Evidence for genetic exchange with cyanobacteria. Applied and environmental microbiology. 2013:AEM. 03821–12. 10.1128/AEM.03821-12 PMC3697557 23603674

[32] MacGregor BJ, Biddle JF, Harbort C, Matthysse AG, Teske A. Sulfide oxidation, nitrate respiration, carbon acquisition, and electron transport pathways suggested by the draft genome of a single orange Guaymas Basin Beggiatoa (Cand. Maribeggiatoa) sp. filament. Marine genomics. 2013;11:53–65. doi: 10.1016/j.margen.2013.08.001 24012537 10.1016/j.margen.2013.08.001

[33] Kojima H, Ogura Y, Yamamoto N, Togashi T, Mori H, Watanabe T, et al Ecophysiology of Thioploca ingrica as revealed by the complete genome sequence supplemented with proteomic evidence. The ISME journal. 2015;9(5):1166–76. doi: 10.1038/ismej.2014.209 25343513 10.1038/ismej.2014.209 PMC4409161

[34] Winkel M, Salman-Carvalho V, Woyke T, Richter M, Schulz-Vogt HN, Flood BE, et al Single-cell sequencing of Thiomargarita reveals genomic flexibility for adaptation to dynamic redox conditions. Frontiers in microbiology. 2016;7. 10.3389/fmicb.2016.00964 PMC4914600 27446006

[35] Flood BE, Fliss P, Jones DS, Dick GJ, Jain S, Kaster A-K, et al Single-cell (Meta-) genomics of a dimorphic Candidatus Thiomargarita nelsonii reveals genomic plasticity. Frontiers in microbiology. 2016;7. 10.3389/fmicb.2016.00603 PMC4853749 27199933

[36] Salman-Carvalho V, Fadeev E, Joye SB, Teske A. How Clonal Is Clonal? Genome Plasticity across Multicellular Segments of a “Candidatus Marithrix sp.” Filament from Sulfidic, Briny Seafloor Sediments in the Gulf of Mexico. Frontiers in Microbiology. 2016;7. 10.3389/fmicb.2016.01173 PMC4971068 27536274

[37] Fomenkov A, Vincze T, Grabovich MY, Dubinina G, Orlova M, Belousova E, et al Complete genome sequence of the freshwater colorless sulfur bacterium Beggiatoa leptomitiformis neotype strain D-402T. Genome announcements. 2015;3(6). 10.1128/genomeA.01436-15 PMC4675945 26659680

[38] Sobarzo M, Djurfeldt L. Coastal upwelling process on a continental shelf limited by submarine canyons, Concepción, central Chile. Journal of Geophysical Research: Oceans. 2004;109(C12).

[39] Ishoey T, Woyke T, Stepanauskas R, Novotny M, Lasken RS. Genomic sequencing of single microbial cells from environmental samples. Current opinion in microbiology. 2008;11(3):198–204. doi: 10.1016/j.mib.2008.05.006 18550420 10.1016/j.mib.2008.05.006 PMC3635501

[40] Myers EW, Sutton GG, Delcher AL, Dew IM, Fasulo DP, Flanigan MJ, et al A whole-genome assembly of Drosophila. Science. 2000;287(5461):2196–204. 10731133 10.1126/science.287.5461.2196

[41] Strous M, Kraft B, Bisdorf R, Tegetmeyer HE. The binning of metagenomic contigs for microbial physiology of mixed cultures. Frontiers in microbiology. 2012;3 doi: 10.3389/fmicb.2012.00003 23227024 10.3389/fmicb.2012.00410 PMC3514610

[42] Wu M, Scott AJ. Phylogenomic analysis of bacterial and archaeal sequences with AMPHORA2. Bioinformatics. 2012;28(7):1033–4. doi: 10.1093/bioinformatics/bts079 22332237 10.1093/bioinformatics/bts079

[43] Campbell JH, O’Donoghue P, Campbell AG, Schwientek P, Sczyrba A, Woyke T, et al UGA is an additional glycine codon in uncultured SR1 bacteria from the human microbiota. Proceedings of the National Academy of Sciences. 2013;110(14):5540–5. 10.1073/pnas.1303090110 PMC3619370 23509275

[44] Aziz RK, Bartels D, Best AA, DeJongh M, Disz T, Edwards RA, et al The RAST Server: rapid annotations using subsystems technology. BMC genomics. 2008;9(1):75. 18261238 10.1186/1471-2164-9-75 PMC2265698

[45] Seemann T. Prokka: rapid prokaryotic genome annotation. Bioinformatics. 2014:btu153. 10.1093/bioinformatics/btu153 24642063

[46] Finn RD, Tate J, Mistry J, Coggill PC, Sammut SJ, Hotz H-R, et al The Pfam protein families database. Nucleic acids research. 2008;36(suppl 1):D281–D8. 18039703 10.1093/nar/gkm960 PMC2238907

[47] Medema MH, Blin K, Cimermancic P, de Jager V, Zakrzewski P, Fischbach MA, et al antiSMASH: rapid identification, annotation and analysis of secondary metabolite biosynthesis gene clusters in bacterial and fungal genome sequences. Nucleic acids research. 2011;39(suppl 2):W339–W46. 21672958 10.1093/nar/gkr466 PMC3125804

[48] Blin K, Medema MH, Kazempour D, Fischbach MA, Breitling R, Takano E, et al antiSMASH 2.0—a versatile platform for genome mining of secondary metabolite producers. Nucleic acids research. 2013:gkt449. 10.1093/nar/gkt449 PMC3692088 23737449

[49] Weber T, Blin K, Duddela S, Krug D, Kim HU, Bruccoleri R, et al antiSMASH 3.0—a comprehensive resource for the genome mining of biosynthetic gene clusters. Nucleic acids research. 2015;43(W1):W237–W43. doi: 10.1093/nar/gkv437 25948579 10.1093/nar/gkv437 PMC4489286

[50] Ziemert N, Podell S, Penn K, Badger JH, Allen E, Jensen PR. The natural product domain seeker NaPDoS: a phylogeny based bioinformatic tool to classify secondary metabolite gene diversity. PLoS One. 2012;7(3):e34064 doi: 10.1371/journal.pone.0034064 22479523 10.1371/journal.pone.0034064 PMC3315503

[51] Grissa I, Vergnaud G, Pourcel C. CRISPRFinder: a web tool to identify clustered regularly interspaced short palindromic repeats. Nucleic acids research. 2007;35(suppl 2):W52–W7. 17537822 10.1093/nar/gkm360 PMC1933234

[52] Schatz MC, Phillippy AM, Sommer DD, Delcher AL, Puiu D, Narzisi G, et al Hawkeye and AMOS: visualizing and assessing the quality of genome assemblies. Briefings in bioinformatics. 2013;14(2):213–24. doi: 10.1093/bib/bbr074 22199379 10.1093/bib/bbr074 PMC3603210

[53] Rutherford K, Parkhill J, Crook J, Horsnell T, Rice P, Rajandream M-A, et al Artemis: sequence visualization and annotation. Bioinformatics. 2000;16(10):944–5. 11120685 10.1093/bioinformatics/16.10.944

[54] Krzywinski M, Schein J, Birol I, Connors J, Gascoyne R, Horsman D, et al Circos: an information aesthetic for comparative genomics. Genome research. 2009;19(9):1639–45. doi: 10.1101/gr.092759.109 19541911 10.1101/gr.092759.109 PMC2752132

[55] Huelsenbeck JP, Ronquist F. MRBAYES: Bayesian inference of phylogenetic trees. Bioinformatics. 2001;17(8):754–5. 11524383 10.1093/bioinformatics/17.8.754

[56] Katoh K, Misawa K, Kuma Ki, Miyata T. MAFFT: a novel method for rapid multiple sequence alignment based on fast Fourier transform. Nucleic acids research. 2002;30(14):3059–66. 12136088 10.1093/nar/gkf436 PMC135756

[57] Woese CR, Fox GE, Zablen L, Uchida T, Bonen L, Pechman K, et al Conservation of primary structure in 16S ribosomal RNA. Nature. 1975;254(5495):83–6. 1089909 10.1038/254083a0

[58] FOX GE, Pechman KR, Woese CR. Comparative cataloging of 16S ribosomal ribonucleic acid: molecular approach to procaryotic systematics. International Journal of Systematic and Evolutionary Microbiology. 1977;27(1):44–57.

[59] Fox Gc-a, Stackebrandt E, Hespell R, Gibson J, Maniloff J, Dyer T, et al The phylogeny of prokaryotes. Science. 1980;209(4455):457–63. 6771870 10.1126/science.6771870

[60] Ludwig W, Schleifer K. Bacterial phylogeny based on 16S and 23S rRNA sequence analysis. FEMS microbiology reviews. 1994;15(2–3):155–73. 7524576 10.1111/j.1574-6976.1994.tb00132.x

[61] Nelson DC, Waterbury JB, Jannasch HW. Nitrogen fixation and nitrate utilization by marine and freshwater Beggiatoa. Archives of Microbiology. 1982;133(3):172–7.

[62] de Albuquerque JP, Keim CN, Lins U. Comparative analysis of Beggiatoa from hypersaline and marine environments. Micron. 2010;41(5):507–17. doi: 10.1016/j.micron.2010.01.009 20207153 10.1016/j.micron.2010.01.009

[63] Kamp A, Røy H, Schulz-Vogt HN. Video-supported analysis of Beggiatoa filament growth, breakage, and movement. Microbial Ecology. 2008;56(3):484–91. doi: 10.1007/s00248-008-9367-x 18335158 10.1007/s00248-008-9367-x PMC2755761

[64] Grünke S, Lichtschlag A, Beer Dd, Felden J, Salman V, Ramette A, et al Mats of psychrophilic thiotrophic bacteria associated with cold seeps of the Barents Sea. Biogeosciences. 2012;9(8):2947–60.

[65] Stackebrandt E, Goebel B. Taxonomic note: a place for DNA-DNA reassociation and 16S rRNA sequence analysis in the present species definition in bacteriology. International Journal of Systematic and Evolutionary Microbiology. 1994;44(4):846–9.

[66] Schloss PDH, Jo. DOTUR, Introducing a computer program for defining operational taxonomic units and estimating species richness Applied and Environmental Microbiology. 2005;71(3):1501–6. doi: 10.1128/AEM.71.3.1501-1506.2005 15746353 10.1128/AEM.71.3.1501-1506.2005 PMC1065144

[67] Maier S, Gallardo V. Thioploca araucae sp. nov. and Thioploca chileae sp. nov. International Journal of Systematic and Evolutionary Microbiology. 1984;34(4):414–8.

[68] Maier S, Völker H, Beese M, Gallardo VA. The fine structure of Thioploca araucae and Thioploca chileae. Canadian Journal of Microbiology. 1990;36(6):438–48.

[69] McHatton SC, Barry JP, Jannasch HW, Nelson DC. High nitrate concentrations in vacuolate, autotrophic marine Beggiatoa spp. Applied and Environmental Microbiology. 1996;62(3):954–8. 16535282 10.1128/aem.62.3.954-958.1996 PMC1388807

[70] Ahmad A, Barry JP, Nelson DC. Phylogenetic Affinity of a Wide, Vacuolate, Nitrate-AccumulatingBeggiatoa sp. from Monterey Canyon, California, withThioploca spp. Applied and environmental microbiology. 1999;65(1):270–7. 9872789 10.1128/aem.65.1.270-277.1999 PMC91012

[71] Papaspyrou S, Smith CJ, Dong LF, Whitby C, Dumbrell AJ, Nedwell DB. Nitrate reduction functional genes and nitrate reduction potentials persist in deeper estuarine sediments. Why? PloS one. 2014;9(4):e94111 doi: 10.1371/journal.pone.0094111 24728381 10.1371/journal.pone.0094111 PMC3984109

[72] Bell LC, Richardson DJ, Ferguson SJ. Periplasmic and membrane-bound respiratory nitrate reductases in Thiosphaera pantotropha: the periplasmic enzyme catalyzes the first step in aerobic denitrification. FEBS letters. 1990;265(1–2):85–7. 2365057 10.1016/0014-5793(90)80889-q

[73] Li Y, Katzmann E, Borg S, Schüler D. The periplasmic nitrate reductase Nap is required for anaerobic growth and involved in redox control of magnetite biomineralization in Magnetospirillum gryphiswaldense. Journal of bacteriology. 2012;194(18):4847–56. doi: 10.1128/JB.00903-12 22730130 10.1128/JB.00903-12 PMC3430331

[74] Einsle O. Structure and function of formate-dependent cytochrome c nitrite reductase, NrfA. Methods Enzymol. 2011;496:399–422. doi: 10.1016/B978-0-12-386489-5.00016-6 21514473 10.1016/B978-0-12-386489-5.00016-6

[75] Simon J, Gross R, Einsle O, Kroneck PM, Kröger A, Klimmek O. A NapC/NirT‐type cytochrome c (NrfH) is the mediator between the quinone pool and the cytochrome c nitrite reductase of Wolinella succinogenes. Molecular microbiology. 2000;35(3):686–96. 10672190 10.1046/j.1365-2958.2000.01742.x

[76] Gallardo V. On the discovery of a large microbial community living in the soft bottoms of the continental shelf of Chile and Peru. Anales del Instituto de Investigaciones Marinas, Pta Betín (Colombia)(Supplemento 1). 1977:23–30.

[77] Teske A, Nelson DC. The genera Beggiatoa and Thioploca. The prokaryotes: Springer; 2006 p. 784–810.

[78] Brinkhoff T, Kuever J, Muyzer G, Jannasch HW. Thiomicrospira. Bergey's Manual of Systematics of Archaea and Bacteria. 2005.

[79] Durand P, Reysenbach A-L, Prieur D, Pace N. Isolation and characterization of Thiobacillus hydrothermalis sp. nov., a mesophilic obligately chemolithotrophic bacterium isolated from a deep-sea hydrothermal vent in Fiji Basin. Archives of microbiology. 1993;159(1):39–44.

[80] Stockdreher Y, Venceslau SS, Josten M, Sahl H-G, Pereira IA, Dahl C. Cytoplasmic sulfurtransferases in the purple sulfur bacterium Allochromatium vinosum: evidence for sulfur transfer from DsrEFH to DsrC. PLoS One. 2012;7(7):e40785 doi: 10.1371/journal.pone.0040785 22815818 10.1371/journal.pone.0040785 PMC3397948

[81] Dubinina G, Savvichev A, Orlova M, Gavrish E, Verbarg S, Grabovich M. Beggiatoa leptomitoformis sp. nov., the first freshwater member of the genus capable of chemolithoautotrophic growth. International journal of systematic and evolutionary microbiology. 2017;67(2):197–204. doi: 10.1099/ijsem.0.001584 27902215 10.1099/ijsem.0.001584

[82] Hedderich R, Hamann N, Bennati M. Heterodisulfide reductase from methanogenic archaea: a new catalytic role for an iron-sulfur cluster. Biological chemistry. 2005;386(10):961–70. doi: 10.1515/BC.2005.112 16218868 10.1515/BC.2005.112

[83] Stojanowic A, Mander GJ, Duin EC, Hedderich R. Physiological role of the F420-non-reducing hydrogenase (Mvh) from Methanothermobacter marburgensis. Archives of microbiology. 2003;180(3):194–203. doi: 10.1007/s00203-003-0577-9 12856108 10.1007/s00203-003-0577-9

[84] Friedrich CG, Bardischewsky F, Rother D, Quentmeier A, Fischer J. Prokaryotic sulfur oxidation. Current opinion in microbiology. 2005;8(3):253–9. doi: 10.1016/j.mib.2005.04.005 15939347 10.1016/j.mib.2005.04.005

[85] Wall J, Arkin A, Rapp-Giles B. Genetics and genomics of sulfate respiration in Desulfovibrio, p 1–12. Microbial sulfur metabolism Springer-Verlag, Heidelberg, Germany 2008.

[86] Dahl C. Inorganic sulfur compounds as electron donors in purple sulfur bacteria Sulfur metabolism in phototrophic organisms: Springer; 2008 p. 289–317.

[87] Hensen D, Sperling D, Trüper HG, Brune DC, Dahl C. Thiosulphate oxidation in the phototrophic sulphur bacterium Allochromatium vinosum. Molecular microbiology. 2006;62(3):794–810. doi: 10.1111/j.1365-2958.2006.05408.x 16995898 10.1111/j.1365-2958.2006.05408.x

[88] Dahl C, Prange A. Bacterial sulfur globules: occurrence, structure and metabolism. Inclusions in prokaryotes: Springer; 2006 p. 21–51.

[89] Dahl C, Friedrich CG. Microbial sulfur metabolism: Springer; 2008.

[90] Muntyan M, Grabovich MY, Patritskaya VY, Dubinina G. Regulation of metabolic and electron transport pathways in the freshwater bacterium Beggiatoa leptomitiformis D-402. Microbiology. 2005;74(4):388–94. 16211847

[91] Lee PA, De Mora SJ. Intracellular dimethylsulfoxide (DMSO) in unicellular marine algae: speculations on its origin and possible biological role. Journal of Phycology. 1999;35(1):8–18.

[92] Escribano R, Daneri G, Farías L, Gallardo VA, González HE, Gutiérrez D, et al Biological and chemical consequences of the 1997–1998 El Niño in the Chilean coastal upwelling system: a synthesis. Deep Sea Research Part II: Topical Studies in Oceanography. 2004;51(20):2389–411.

[93] Wood AP, Aurikko JP, Kelly DP. A challenge for 21st century molecular biology and biochemistry: what are the causes of obligate autotrophy and methanotrophy? FEMS microbiology reviews. 2004;28(3):335–52. 15449607 10.1016/j.femsre.2003.12.001

[94] Hagen KD, Nelson DC. Organic carbon utilization by obligately and facultatively autotrophic beggiatoa strains in homogeneous and gradient cultures. Applied and environmental microbiology. 1996;62(3):947–53. 16535281 10.1128/aem.62.3.947-953.1996 PMC1388806

[95] Schwedt A, Kreutzmann A-C, Polerecky L, Schulz-Vogt HN. Sulfur respiration in a marine chemolithoautotrophic Beggiatoa strain. Frontiers in microbiology. 2012;2:1–8. 10.3389/fmicb.2011.00276 PMC3253548 22291687

[96] Burnett WC. Phosphorite deposits from the sea floor off Peru and Chile: radiochemical and geochemical investigations concerning their origin 1974.

[97] Motomura K, Hirota R, Okada M, Ikeda T, Ishida T, Kuroda A. A new subfamily of polyphosphate kinase 2 (class III PPK2) catalyzes both nucleoside monophosphate phosphorylation and nucleoside diphosphate phosphorylation. Applied and environmental microbiology. 2014;80(8):2602–8. doi: 10.1128/AEM.03971-13 24532069 10.1128/AEM.03971-13 PMC3993171

[98] Akiyama M, Crooke E, Kornberg A. An exopolyphosphatase of Escherichia coli. The enzyme and its ppx gene in a polyphosphate operon. Journal of Biological Chemistry. 1993;268(1):633–9. 8380170

[99] Makarova KS, Haft DH, Barrangou R, Brouns SJ, Charpentier E, Horvath P, et al Evolution and classification of the CRISPR–Cas systems. Nature Reviews Microbiology. 2011;9(6):467–77. doi: 10.1038/nrmicro2577 21552286 10.1038/nrmicro2577 PMC3380444

[100] Makarova KS, Koonin EV. Annotation and classification of CRISPR-Cas systems. CRISPR: Methods and Protocols. 2015:47–75. 10.1007/978-1-4939-2687-9_4 PMC5901762 25981466

[101] Makarova KS, Wolf YI, Alkhnbashi OS, Costa F, Shah SA, Saunders SJ, et al An updated evolutionary classification of CRISPR-Cas systems. Nature Reviews Microbiology. 2015. 10.1038/nrmicro3569 PMC5426118 26411297

[102] Sinkunas T, Gasiunas G, Fremaux C, Barrangou R, Horvath P, Siksnys V. Cas3 is a single‐stranded DNA nuclease and ATP‐dependent helicase in the CRISPR/Cas immune system. The EMBO journal. 2011;30(7):1335–42. doi: 10.1038/emboj.2011.41 21343909 10.1038/emboj.2011.41 PMC3094125

[103] Rollins MF, Schuman JT, Paulus K, Bukhari HS, Wiedenheft B. Mechanism of foreign DNA recognition by a CRISPR RNA-guided surveillance complex from Pseudomonas aeruginosa. Nucleic acids research. 2015;43(4):2216–22. doi: 10.1093/nar/gkv094 25662606 10.1093/nar/gkv094 PMC4344526

[104] Heussler GE, Miller JL, Price CE, Collins AJ, O'Toole GA. Requirements for Pseudomonas aeruginosa Type IF CRISPR-Cas Adaptation Determined Using a Biofilm Enrichment Assay. Journal of Bacteriology. 2016;198(22):3080–90. doi: 10.1128/JB.00458-16 27573013 10.1128/JB.00458-16 PMC5075037

[105] Li X, Vanner S, Wang W, Li Y, Gallardo VA, Magarvey NA. Macplocimine A, a new 18-membered macrolide isolated from the filamentous sulfur bacteria Thioploca sp. The Journal of antibiotics. 2013;66(7):443–6. doi: 10.1038/ja.2013.52 23778115 10.1038/ja.2013.52

[106] Gershenzon J, Dudareva N. The function of terpene natural products in the natural world. Nature chemical biology. 2007;3(7):408–14. doi: 10.1038/nchembio.2007.5 17576428 10.1038/nchembio.2007.5

[107] Yamada Y, Kuzuyama T, Komatsu M, Shin-ya K, Omura S, Cane DE, et al Terpene synthases are widely distributed in bacteria. Proceedings of the National Academy of Sciences. 2015;112(3):857–62. 10.1073/pnas.1422108112 PMC4311827 25535391

[108] Alcaíno J, Baeza M, Cifuentes V. Carotenoid Distribution in Nature. Carotenoids in Nature: Springer; 2016 p. 3–33. 10.1007/978-3-319-39126-7_1 27485217

