# Supplementary material for: Genomic features of “Candidatus Venteria ishoeyi”, a new sulfur-oxidizing macrobacterium from the Humboldt Sulfuretum off Chile
Source: PLoS One. 2017 Dec 13;12(12):e0188371. doi: 10.1371/journal.pone.0188371 (PMC5728499; doi:10.1371/journal.pone.0188371)
Supplement: S1 Fig — The top of the figure shows the amplification products of the full length (~1.5 kbp) 16S rDNA PCR products of the seven micro-manipulated bacterial filaments (MDA1—MDA7). The PCR products were purified and sequenced using 27F and 1492R primers by Sanger sequencing and visualized by agarose gel electrophoresis (1%) E-gels (Invitrogen) with a low DNA mass 2kb ladder. The bottom of the figure (MDA-DNA) shows the products of whole genome amplification (WGA) of the single filaments MDA1 to MDA7, and the MDA no template control (NTC) visualized through agarose gel electrophoresis (1%) E-gels (Invitrogen). (PDF) [file pone.0188371.s001.pdf]

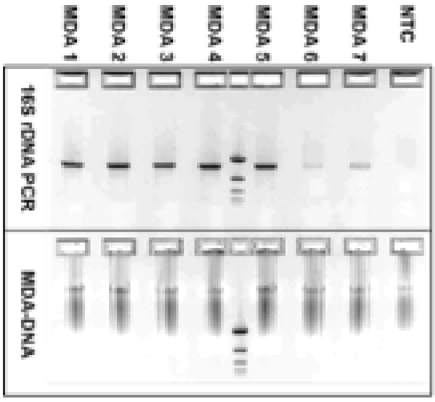

S1 Fig. Amplification products of 16S rDNA genes and whole genome amplification (WGA) from seven bacterial filaments (MDA1-7) isolated by micromanipulation and visualized using agarose gel electrophoresis. The top of the figure shows the amplification products of the full length (~1.5kbp) 16S rDNA PCR products of the seven micro-manipulated bacterial filaments (MDA1 - MDA7). The PCR products were purified and sequenced using 27F and 1492R primers by Sanger sequencing and visualized by agarose gel electrophoresis (1%) E-gels (Invitrogen) with a low DNA mass 2kb ladder. The bottom of the figure (MDA-DNA) shows the products of whole genome amplification (WGA) of the single filaments MDA1 to MDA7 and the MDA no template control (NTC) visualized through agarose gel electrophoresis (1%) E-gels (Invitrogen).
