# Supplementary figures and images for: Genomic features of “Candidatus Venteria ishoeyi”, a new sulfur-oxidizing macrobacterium from the Humboldt Sulfuretum off Chile
Source: PLoS One. 2017 Dec 13;12(12):e0188371. doi: 10.1371/journal.pone.0188371 (PMC5728499; doi:10.1371/journal.pone.0188371)

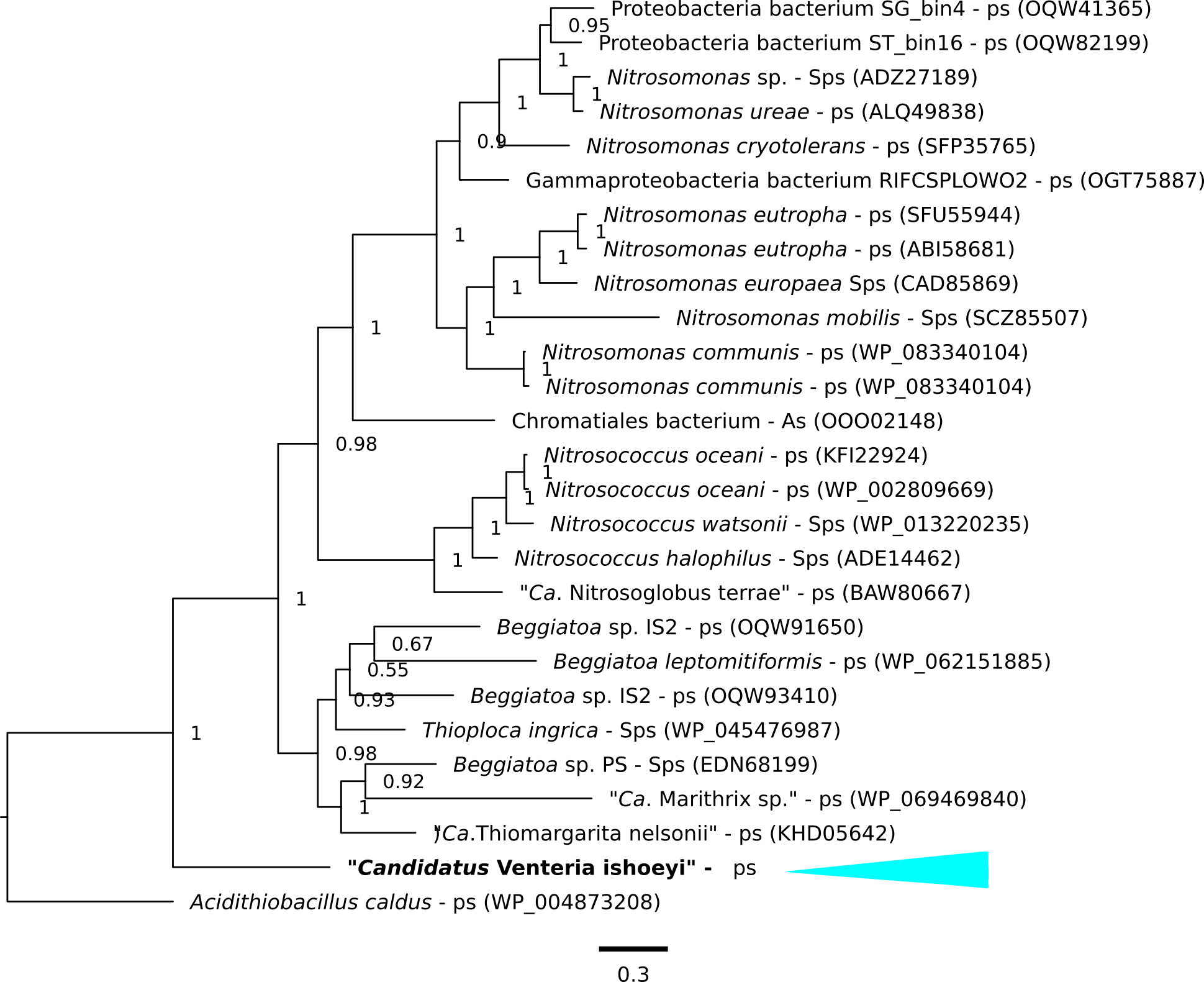

Supplement: S2 Fig — The aligning of the amino acid sequence of the phytoene synthase (ps) identified in “Ca. V. ishoeyi” and 25 amino acid sequences of the Squalene/Phytoene synthase (Sps) and Phytoene synthases taken from the top 50 blastp match, and a sequence of Acidithiobacillus caldus (WP_004873208) as root, was used to build the phylogenetic tree. (TIF) [file pone.0188371.s002.tif]
