# Supplementary material for: Genomic features of “Candidatus Venteria ishoeyi”, a new sulfur-oxidizing macrobacterium from the Humboldt Sulfuretum off Chile
Source: PLoS One. 2017 Dec 13;12(12):e0188371. doi: 10.1371/journal.pone.0188371 (PMC5728499; doi:10.1371/journal.pone.0188371)
Supplement: S1 Table — (PDF) [file pone.0188371.s003.pdf]

S1 Table. **Identification of amplified filaments by SeqMatch function (Ribosomal Database Project, RDPII)**.

Date	Query name	S_ab_score	Sequence name (RDP SeqMatch to cultured isolates)	Form
12/8/08	MDA1*	0,615	*"Beggiatoa"* sp. MS-81-1c;AF1102276	Gammaproteobacterium
12/8/08	MDA2*	0,617	*"Beggiatoa"* sp. MS-81-1c;AF1102276	Gammaproteobacterium
12/8/08	MDA3	0,797	*Maorithyas hadalis* gill thioautotrophic symbiont II; AB188780	Gammaproteobacterium
12/8/08	MDA5*	0,613	*"Beggiatoa"*sp. MS-81-1c;AF1102276	Gammaproteobacterium
12/8/08	MDA6np	0,615	*Desulfonema magnum* (T); DSM2077	Deltaproteobacterium

Asterisks indicate the MDA-DNAs selected to be sequenced by 454 GS FLX technology.
