# Supplementary material for: Genomic features of “Candidatus Venteria ishoeyi”, a new sulfur-oxidizing macrobacterium from the Humboldt Sulfuretum off Chile
Source: PLoS One. 2017 Dec 13;12(12):e0188371. doi: 10.1371/journal.pone.0188371 (PMC5728499; doi:10.1371/journal.pone.0188371)
Supplement: S2 Table — (PDF) [file pone.0188371.s004.pdf]

S2 Table. Genes coding for sulfur oxidation enzymes in “*Ca*. V. ishoeyi”.

Abbreviation	Enzyme	Gene	Locus tag	Scaffold	CDS start	CDS end
Fcc	Flavocytochrome c/sulfide dehydrogenase	fccA	MBHS_04615	scf_492	4803	5120
		fccA	MBHS_04676	scf_492	69279	69596
		fccB	MBHS_04616	scf_492	5130	6425
		fccB	MBHS_04675	scf_492	68727	69269
Sqr	Sulfide:quinone oxido-reductase	sqr	MBHS_00764	scf_126	28830	29954
Sox	Thiosulfate-oxidizing Sox enzyme system	soxAX	MBHS_04050	scf_485	10893	11720
		soxAX	MBHS_04079	scf_486	12880	13710
		soxY	MBHS_04077	scf_486	11826	12290
		soxZ	MBHS_04078	scf_486	12396	12710
Dsr	Dissimilatory sulfite reductase system	dsrA	MBHS_03945	scf_483	96302	97564
		dsrB	MBHS_03946	scf_483	97627	98691
		dsrE	MBHS_03947	scf_483	98711	99103
		dsrF	MBHS_03948	scf_483	99205	99621
		dsrH	MBHS_03949	scf_483	99635	99940
		dsrC	MBHS_03950	scf_483	100001	100336
		dsrM	MBHS_03951	scf_483	100464	101213
		dsrK	MBHS_03952	scf_483	101238	102764
		dsrL	MBHS_03953	scf_483	102861	104804
		dsrJ	MBHS_03954	scf_483	104882	105409
		dsrO	MBHS_03955	scf_483	105406	106161
		dsrP	MBHS_03956	scf_483	106172	107377
Apr	Adenylylsulfate reductase (APS reductase)	aprA	MBHS_04595	scf_491	176146	178143
		aprB	MBHS_04596	scf_491	178235	178708
Hdr	Heterodisulfide reductase	hdrA	MBHS_00323	scf_51	50915	51511
		hdrB	MBHS_00322	scf_51	50254	50865
Sat	Sulfate adenylyltransferase	sat	MBHS_00245	scf_47	46029	47222
