# Supplementary material for: Genomic features of “Candidatus Venteria ishoeyi”, a new sulfur-oxidizing macrobacterium from the Humboldt Sulfuretum off Chile
Source: PLoS One. 2017 Dec 13;12(12):e0188371. doi: 10.1371/journal.pone.0188371 (PMC5728499; doi:10.1371/journal.pone.0188371)
Supplement: S3 Table — In the tricarboxylic acid cycle; S3B. Involved in glycolysis; S3C. Involved in the Calvin-Benson-Bassham cycle. (PDF) [file pone.0188371.s005.pdf]

S3A Table. Genes involved in the Tricarboxylic acid cycle identified in "*Ca* V. ishoeyi".

Tricarboxylic acid cycle				
Product	Gene	Locus	Scaffold ID	Protein sequence length
Citrate synthase (type I and II)	gltA	Not in "*Ca*Vi"	n.d	n.d
Citrate synthase	aarA	MBHS_02352	scf_441	432
Aconitate hydratase I and II	acnA/acnB	MBHS_02556	scf_450	852
Isocitrate dehydrogenase	icd	MBHS_03490	scf_477	292
2-oxoglutarate dehydrogenase E1	sucA	MBHS_00167	scf_042	970
Dihydrolipoyl succinyltransferase E2	sucB	MBHS_01480<br>MBHS_04108	scf_339<br>scf_487	93<br>531
Succinyl-CoA synthetase, beta subunit	sucC	MBHS_04498	scf_491	390
Succinyl-CoA synthetase, alpha subunit	sucD	MBHS_04499	scf_491	290
Succinate dehydrogenase/ fumarate reductase, flavoprotein subunit	sdhA	MBHS_00714<br>MBHS_03266	Scf_123<br>scf_473	128<br>598
Succinate dehydrogenase/ fumarate reductase, iron-sulfur subunit	sdhB	MBHS_00390	scf_553	259
Succinate dehydrogenase/ fumarate reductase, cytochrome b556 subunit	sdhC	MBHS_03220	scf_472	126
Succinate dehydrogenase/ fumarate reductase, membrane anchor	sdhD	Not in "*Ca*Vi"	n.d	n.d
Fumarate hydratase	fumAB<br>(fumB)	MBHS_00665	scf_118	501
Malate dehydrogenase	mlate<br>(mdh)	MBHS_01386<br>MBHS_01427<br>MBHS_02883	scf_330<br>scf_334<br>scf_464	327<br>323<br>327

N.d : No data.

Between brackets is indicate the synonymous gene name or possible substitute of the common gene in the cycle.

Question marks indicate that the presence of the gene encoding for a product is unknown in "CaVi"'s draft genome.

S3B Table. Genes involved in glycolysis identified in "*Ca* V. ishoeyi".

Glycolysis				
Product	Gene	Locus	Scaffold ID	Length protein sequence
glucokinase	glk	MBHS_01584<br>MBHS_03624	scf_355<br>scf_478	317<br>472
polyphosphate glucokinase	ppgk	Not in "*Ca*Vi"	n.d	n.d
glucose-6-phosphate isomerase	pgi	MBHS_02041	scf_430	543
6-phosphofructokinase, pyrophosphate dependent	pfk	Not in "*Ca*Vi"	n.d	n.d
bisphosphate aldolase A	fbaA	MBHS_02512	scf_452	343
bisphosphate aldolase B	fbaB	MBHS_03550	scf_477	350
glyceraldehyde-3-phosphate dehydrogenase	gapA	MBHS_03668	scf_478	334
phosphoglycerate kinase	pgk	MBHS_03671	scf_481	392
phosphoglycerate mutase	gpmA	MBHS_00405	scf_59	103
enolase	eno	?	n.d	n.d
pyruvate kinase II	pykA	MBHS_03673	scf_478	493
pyruvate dehydrogenase E1 component	aceE	MBHS_02952	scf_465	886
dihydrolipoamide acetyltransferase E2 component	aceF	MBHS_02953	scf_465	452
pyruvate dehydrogenase E1 component, alpha subunit	pdhA	Not in "*Ca*Vi"	1.2.4.1	158
pyruvate dehydrogenase E1 component, beta subunit	pdhB	Not in "*Ca*Vi"	1.2.4.1	190
dihydrolipoamide acetyltransferase E2 component	pdhC	Not in "*Ca*Vi"	2.3.1.12	266
dihydrolipoamide dehydrogenase	lpd	MBHS_01805<br>MBHS_02606	scf_382<br>scf_450	467<br>469

N.d : No data.

Between brackets is indicate the synonymous gene name or possible substitute of the common gene in the cycle.

Question marks indicate that the presence of the gene encoding for a product is unknown in genome of "*Ca* V. ishoeyi".

S3C Table. Genes involved in the Calvin-Benson-Bassham cycle identified in "*Ca* V. ishoeyi".

Calvin-Benson-Bassham cycle				
Product	Gene	Locus	Scaffold ID	Length protein sequence
Ribulose bisphosphate carboxylase large chain, form I	rbcL	?	n.d	n.d
Ribulose bisphosphate carboxylase large chain, form II	rbcM	?	n.d	n.d.
Ribulose bisphosphate carboxylase	cbbM	MBHS_00487	scf_74	314
Ribulose bisphosphate carboxylase small subunit	rbcS	Not in "*Ca*Vi"	n.d	n.d
Phosphoglycerate kinase	pgk	MBHS_03672	scf_478	392
Fructose-1,6-bisphosphatase	fbp	Not in "*Ca*Vi"	n.d	n.d
Transketolase	tkt (tktA)	MBHS_03667	scf_47	657
Pyrphosphate dependent 6-phosphofructokinase	ppi-PFK	MBHS_03099	scf_468	419
Ribose-5-phosphatisomerase	cbbI (rpiA)	MBHS_04205	scf_489	218
Phosphoribulokinase	PrkA (cfxp)	MBHS_00162	scf_42	295

N.d : No data.

Between brackets is indicate the synonymous gene name or possible substitute of the common gene in the cycle.

Question marks indicate that the presence of the gene encoding for a product is unknown in genome of "*Ca* V. ishoeyi".
