# Supplementary material for: Genomic features of “Candidatus Venteria ishoeyi”, a new sulfur-oxidizing macrobacterium from the Humboldt Sulfuretum off Chile
Source: PLoS One. 2017 Dec 13;12(12):e0188371. doi: 10.1371/journal.pone.0188371 (PMC5728499; doi:10.1371/journal.pone.0188371)
Supplement: S4 Table — S4A. List of CRISPR repeats identified in “Ca. V. ishoeyi”. The table shows: scaffolds containing start and end of CRISPR repeat regions, Direct Repeat (DR) sequences, length of the DR, and number of spacers. S4B. CRISPR-associated protein encoding genes identified in “Ca. V. ishoeyi”. (PDF) [file pone.0188371.s006.pdf]

S4A Table. List of CRISPR repeats identified in "*Ca*. V. ishoeyi". The table shows: scaffolds containing start and end of CRISPR repeat regions, Direct Repeat (DR) sequences, length of the DR, and number of spacers.

Scaffold	Crispr begin position	Crispr end position	Direct Repeat	Length	Number of spacers
Scf_79	5283	6218	1 – GTTTCAGGCATGCTGGCCGCTAAGGCTATTGAAAC	35	14
Scf_137	45	744	2 – ATGTTCCCCACGCCAGTGGGGATGAACCG	29	12
Scf_146	1760	2037	3 – TCTGTTGACGATTTAGATTGTAAAAA	26	4
Scf_160	23	699	4 – GTCTCAATAGCCTAAGCGGCTGACCCTGCTGCAAC	35	10
Scf_160	7256	7435	5 – GTCTCAATAGCCTAAGCGGCTGACCCTGCTGCAAC	35	3
Scf_179	2	1190	6 – CGGTTCATCCCCACTGGCGTGGGGAACAT	29	20
Scf_351	6361	7169	7 – TTTCTAAGCTGCCTGTGCGGCAGTCAAC	28	14
Scf_355	7	514	8 – TTTCTAAGCTGCCTGTGCGGCAGTCAAC	28	9
Scf_355	69212	71101	9 – TTTCTAAGCTGCCTGTGCGGCAGTCAAC	28	32
Scf_422	530	908	10 – GTTTCAATAGCCTTAGCGGCCAGCATGCCTGAAAC	35	6
Scf_456	31078	32520	11 – GTTACGAATCCCCATGAGGGGTTATGAG	28	23
Scf_464	84239	84449	12 – GAAACACCCCCACGGGCGTGGGGAAGAC	28	4
Scf_473	44	3276	13 – GTTACGAATCCCCATGAGGGGTTATGAG	28	51
Scf_479	18897	19618	14 – GTTTCAATAGCCTTAGCGGCCAGCATGCCTGAAAC	35	11
Scf_483	110535	111464	15 – GTTGACTGCCGCACAGGCAGCTTAGAAA	28	16
Scf_483	112279	118854	16 – GTTGACTGCCGCACAGGCAGCTTAGAAA	28	110
Scf_490	114080	115160	17 – GTTTCAGACGCGTTGGCCGTTTAGGCTGTTGAAAC	35	16
Scf_490	121113	121928	18 – GTTTCAGACGCGTTGGCCGCTTAGGCTGTTGAAAC	35	12

S4B Table. CRISPR-associated protein encoding genes, identified in "*Ca*. V. ishoeyi".

Scaffold	Strand	Gene	CDS star	CDS end	ID	Product
Scf_160	-	cas2	967	1245	MBHS_00980	CRISPR-associated endoribonuclease Cas2
Scf_160	-	cas4-cas1	2339	3475	MBHS_00983	CRISPR-associated protein Cas4/endonuclease Cas1 fusion
Scf_160	+	?	8831	9514	MBHS_00987	CRISPR-associated protein
Scf_160	+	?	10785	11822	MBHS_00989	CRISPR-associated protein (Cas_Cmr3)
Scf_160		?	11933	12847	MBHS_00990	RAMP superfamily protein
Scf_160	+	cmr5	12844	13227	MBHS_00991	CRISPR system Cmr subunit Cmr5
Scf_160		?	13220	14398	MBHS_00992	RAMP superfamily protein
Scf_160	+	?	15861	17156	MBHS_00995	CRISPR-associated protein (Cas_Cas02710)
Scf_246	+	cas3	1490	2332	MBHS_01151	CRISPR-associated nuclease/helicase Cas3
Scf_246	+	casA	2680	4266	MBHS_01153	CRISPR system Cascade subunit CasA
Scf_246	+	?	4282	4752	MBHS_01154	CRISPR-associated protein Cse2 (CRISPR_cse2)
Scf_246	+	casC	4765	5832	MBHS_01155	CRISPR system Cascade subunit CasC
Scf_246	+	casD	5777	6631	MBHS_01156	CRISPR system Cascade subunit CasD
Scf_246	+	casE	6618	7253	MBHS_01157	CRISPR system Cascade subunit CasE
Scf_355	-	cas6f	646	1227	MBHS_01570	CRISPR-associated endonuclease Cas6/Csy4
Scf_355	-	csy3	1232	2272	MBHS_01571	CRISPR-associated protein Csy3
Scf_355	-	csy2	2349	3242	MBHS_01572	CRISPR-associated protein Csy2
Scf_355	-	csy1	3239	4483	MBHS_01573	CRISPR-associated protein Csy1
Scf_355	-	cas3	5036	8350	MBHS_01575	CRISPR-associated nuclease/helicase Cas3 subtype I-F/YPEST
Scf_355	-	cas1	8347	9294	MBHS_01576	CRISPR-associated endonuclease Cas1
Scf_355	+	csy3	71387	71665	MBHS_01645	CRISPR-associated protein Csy3
Scf_385	+	cas6	69993	70679	MBHS_01872	CRISPR-associated endonuclease Cas6
Scf_456	+	?	21438	22175	MBHS_02775	CRISPR associated protein Cas6
Scf_456	+	cas1	29452	30420	MBHS_02781	CRISPR-associated endonuclease Cas1
Scf_456	+	cas2	30617	30919	MBHS_02782	CRISPR-associated endoribonuclease Cas2

Scf_479	+	?	3152	5212	MBHS_03732	RAMP superfamily protein
Scf_479	-	?	6742	7470	MBHS_04050	RAMP superfamily protein
Scf_479	+	?	8653	10053	MBHS_03734	RAMP superfamily protein
Scf_479	+	cas4-cas1	13890	16403	MBHS_03740	CRISPR-associated protein Cas4/endonuclease Cas1 fusion
Scf_479	+	cas2b	16400	16678	MBHS_03741	CRISPR-associated endonuclease Cas2 2
Scf_490	+	cas2	120558	120836	MBHS_04318	CRISPR-associated endoribonuclease Cas2
Scf_490	+	?	125509	127728	MBHS_04320	RAMP superfamily protein
Scf_490	+	?	130994	132406	MBHS_04323	RAMP superfamily protein

Question marks indicate that the gene name is unknown for the product coding in the CDS listed.
