# Supplementary material for: Genomic features of “Candidatus Venteria ishoeyi”, a new sulfur-oxidizing macrobacterium from the Humboldt Sulfuretum off Chile
Source: PLoS One. 2017 Dec 13;12(12):e0188371. doi: 10.1371/journal.pone.0188371 (PMC5728499; doi:10.1371/journal.pone.0188371)
Supplement: S5 Table — S5A. Gene and product names of the Terpene type gene cluster. S5B. Gene and product names of the gene cluster, related with the potential biosynthesis of a non-classified secondary metabolite. (PDF) [file pone.0188371.s007.pdf]

S5A Table. The gene and product names of the Terpene type gene cluster.

Gene	Product
gmr	Cyclic di-GMP phosphodiesterase Gmr
crp	cAMP receptor protein
tag-1	no_tag
arcB	Aerobic respiration control sensor protein ArcB
luxR	Transcriptional activator
crtB	All-trans-phytoene synthase
zraS	Sensor protein ZraS
zraR	Transcriptional regulatory protein ZraR
tag-2	Plasmid maintenance system killer protein
yddM	putative HTH-type transcriptional regulator YddM
carA	Carbamoyl-phosphate synthase small chain
ilvB	Acetolactate synthase large subunit
hypB	Hydrogenase isoenzymes nickel incorporation protein HypB
tag-3	Cytochrome c' precursor

S5B Table. The gene and product names of the gene cluster, related with the potentially biosynthesis of a non-classified secondary metabolite.

Gene	Product
tag-1	Hypothetical protein
trxA	Thioredoxin-1
sgpA	Sulfur globule protein CV1 precursor
mdtC	Multidrug resistance protein MdtC
tag-2	Putative efflux pump membrane fusion protein
tag-3	Hypothetical protein

plsC	1-acyl-sn-glycerol-3-phosphate acyltransferase
tag-4	D-glycero-beta-D-manno-heptose-1,7-bisphosphate 7-phosphatase
tag-5	MazG nucleotide pyrophosphohydrolase domain protein
thrC	Threonine synthase
tag-6	Hypothetical protein
tag-7	Recombination protein F
hom	Homoserine dehydrogenase
alaC	Glutamate-pyruvate aminotransferase AlaC
rpsU	30S ribosomal protein S21
tag-8	Yqey-like protein
dnaG	DNA primase
rpoD	RNA polymerase sigma factor RpoD
pksJ	Polyketide synthase PksJ
cysM	Cysteine synthase B
tag-9	Rhomboid family protein
ydaM	Putative diguanylate cyclase YdaM
podJ	Localization factor PodJL
leuS	Leucine--tRNA ligase
tag-10	Hypothetical protein
tag-11	ABC transporter ATP-binding protein
mepM	Murein DD-endopeptidase MepM
gyrB	DNA gyrase subunit B
tag-12	Hypothetical protein
mreB	Rod shape-determining protein MreB
mreC	Cell shape-determining protein MreC
mreD	Rod shape-determining protein MreD

spoVD	Stage V sporulation protein D
arpB	Antibiotic efflux pump membrane transporter ArpB
